# Critical evaluation of human health risks due to hydraulic fracturing in natural gas and petroleum production

**DOI:** 10.1007/s00204-020-02758-7

**Published:** 2020-05-09

**Authors:** Klaus-Michael Wollin, G. Damm, H. Foth, A. Freyberger, T. Gebel, A. Mangerich, U. Gundert-Remy, F. Partosch, C. Röhl, T. Schupp, Jan G. Hengstler

**Affiliations:** 1 Formerly Public Health Agency of Lower Saxony, Hannover, Germany; 2 Department of Hepatobiliary Surgery and Visceral Transplantation, University Hospital, Leipzig University, Leipzig, Germany; 3 Institute of Environmental Toxicology, University of Halle, Halle/Saale, Germany; 4 Research and Development, Translational Sciences-Toxicology, Bayer AG, Wuppertal, Germany; 5 Federal Institute for Occupational Safety and Health, Dortmund, Germany; 6 Molecular Toxicology, Department of Biology, University of Konstanz, Constance, Germany; 7 Institute for Clinical Pharmacology and Toxicology, Charité, Universitätsmedizin Berlin, Berlin, Germany; 8 Institute for Occupational, Social and Environmental Medicine, University Medical Center, Göttingen, Germany; 9 Department of Environmental Health Protection, Schleswig-Holstein State Agency for Social Services, Kiel, Germany; 10 Chemical Engineering, University of Applied Science Muenster, Steinfurt, Germany; 11 Leibniz Research Centre for Working Environment and Human Factors (IfADo), University of Dortmund, Dortmund, Germany

**Keywords:** Hydraulic fracturing, Unconventional natural gas and oil production, Environmental pollution, Human health risk assessment, Epidemiological studies

## Abstract

The use of hydraulic fracturing (HF) to extract oil and natural gas has increased, along with intensive discussions on the associated risks to human health. Three technical processes should be differentiated when evaluating human health risks, namely (1) drilling of the borehole, (2) hydraulic stimulation, and (3) gas or oil production. During the drilling phase, emissions such as NO_*x*_, NMVOCs (non-methane volatile organic compounds) as precursors for tropospheric ozone formation, and SO_*x*_ have been shown to be higher compared to the subsequent phases. In relation to hydraulic stimulation, the toxicity of frac fluids is of relevance. More than 1100 compounds have been identified as components. A trend is to use fewer, less hazardous and more biodegradable substances; however, the use of hydrocarbons, such as kerosene and diesel, is still allowed in the USA. Methane in drinking water is of low toxicological relevance but may indicate inadequate integrity of the gas well. There is a great concern regarding the contamination of ground- and surface water during the production phase. Water that flows to the surface from oil and gas wells, so-called ‘produced water’, represents a mixture of flow-back, the injected frac fluid returning to the surface, and the reservoir water present in natural oil and gas deposits. Among numerous hazardous compounds, produced water may contain bromide, arsenic, strontium, mercury, barium, radioactive isotopes and organic compounds, particularly benzene, toluene, ethylbenzene and xylenes (BTEX). The sewage outflow, even from specialized treatment plants, may still contain critical concentrations of barium, strontium and arsenic. Evidence suggests that the quality of groundwater and surface water may be compromised by disposal of produced water. Particularly critical is the use of produced water for watering of agricultural areas, where persistent compounds may accumulate. Air contamination can occur as a result of several HF-associated activities. In addition to BTEX, 20 HF-associated air contaminants are group 1A or 1B carcinogens according to the IARC. In the U.S., oil and gas production (including conventional production) represents the second largest source of anthropogenic methane emissions. High-quality epidemiological studies are required, especially in light of recent observations of an association between childhood leukemia and multiple myeloma in the neighborhood of oil and gas production sites. In conclusion, (1) strong evidence supports the conclusion that frac fluids can lead to local environmental contamination; (2) while changes in the chemical composition of soil, water and air are likely to occur, the increased levels are still often below threshold values for safety; (3) point source pollution due to poor maintenance of wells and pipelines can be monitored and remedied; (4) risk assessment should be based on both hazard and exposure evaluation; (5) while the concentrations of frac fluid chemicals are low, some are known carcinogens; therefore, thorough, well-designed studies are needed to assess the risk to human health with high certainty; (6) HF can represent a health risk via long-lasting contamination of soil and water, when strict safety measures are not rigorously applied.

## Introduction

**Fig. 1:**
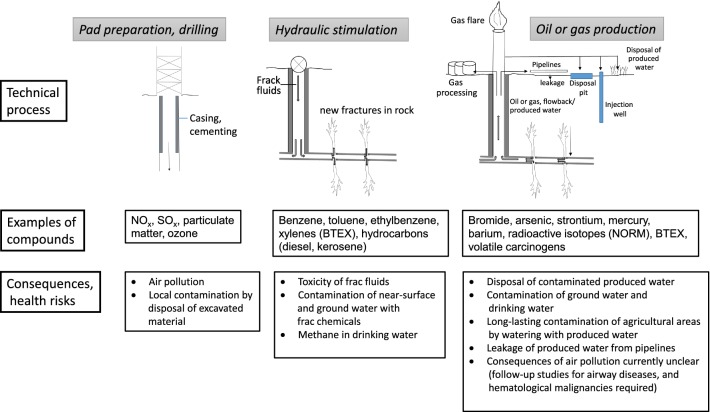
Hydraulic fracturing: the overall process

Hydraulic fracturing (HF) is widely used to enhance oil and gas extraction from source rock and low-permeability shale (U.S. EPA 2016a). This technique is based on the high-pressure injection of a mixture of water, propping agents and frac fluids into a wellbore, with the intention to cause small cracks in oil- or gas-containing deep-rock formations (Fig. 1). The cracks allow an improved flow of oil or gas from their natural reservoirs to the drilling site. HF is required to exploit oil or gas (shale oil or shale gas) from bituminous shale, the so-called ‘non-conventional deposits’. In recent years, HF has become more economically viable because of the development of advanced horizontal drilling techniques in combination with multistage HF, which creates extended fracture networks to enhance the contact area between the rock matrix and the wellbore (Vidic et al. 2013). The U.S. Energy Information Administration (EIA) estimates that in 2018, U.S. dry shale gas production was about 20.95 trillion cubic feet (Tcf) (593.24 × 10^9^ m^3^), and equal to about 69% of total U.S. dry natural gas production in 2018 (U.S. EIA 2019). The U.S. EIA’s “Annual Energy Outlook 2019” predicted an increase of natural gas production as a result of the continued development of tight and shale resources which would account for nearly 90% of dry natural gas production by 2050 (U.S. EIA 2019). China’s shale gas production is predicted to grow from 0.7 billion cubic feet per day (Bcf/day) (19.82 × 10^6^ m^3^/day) in 2016 to 10 Bcf/day (283.17 × 10^6^ m^3^/day) by 2030 and 19 Bcf/day (538.02 × 10^6^ m^3^/day) by 2040 (U.S. EIA 2017). China’s natural gas production from other sources, such as coalbed methane, tight formations and more traditional natural gas reservoirs, is projected to increase more modestly, from 12 Bcf/day (339.80 × 10^6^ m^3^/day) in 2016 to 20 Bcf/day (566.34 × 10^6^ m^3^/day) by 2040.

In Germany, HF has been used to produce gas for more than 4 decades. In Lower Saxony, the German state with the most extensive use of HF, 327 hydraulic stimulations and 148 drillings have been performed, most of which exploited tight gas at depths of at least 3000 m (BGR 2016). Compared to other European countries, Germany has the fourth and fifth largest resources of shale gas and shale oil, respectively. The technically recoverable shale gas resources range between 320 and 2030 billion m^3^ of natural gas at a depth of 1000–5000 m. Including deposits between 500 and 1000 m deep, the total recoverable lean-burn gas quantities have increased to between 380 and 2340 billion m^3^.

The impact of HF on the environment is complex. With respect to human health hazards, the contamination of groundwater and its use as drinking water has been in the center of attention. Moreover, other issues have been raised, such as the high demand for water and land, the impact on biodiversity and landscape, contamination of air by emissions, induced seismic activity, and the greenhouse-gas balance. Due to the rapid development of shale gas extraction, especially in the USA, and the public debate about environmental consequences and human health hazards, possible adverse effects of HF on human health, environmental consequences and the legal frameworks have been discussed (Ewen et al. 2012; Meiners et al. 2012a, b; SRU 2013; Dannwolf et al. 2014; Kersting et al. 2015; U.S. EPA 2016a). The multifaceted topic of HF remains up-to-date against the background of the strong growth of use in the USA and China, the world’s two largest economies. From a Public Health perspective, alone in the USA ~ 17 million residents live within one mile of an active oil and/or gas well and are potentially exposed to pollution as a result of frac operations (U.S. EPA 2016a).

The present review focusses on human health hazards of HF. The technical procedures will be described only to an extent that is necessary to understand toxicological risks for humans. Further aspects, such as energy supply security, influence on ecosystems, biodiversity, landscape, greenhouse gas balance, and socio-economic factors, will not be addressed.

## The procedure of hydraulic fracturing

The six basic steps of HF have already been comprehensively described (e.g., Cheremisinoff and Davletshin 2015; Gandossi and von Estorff 2015; Smith and Montgomery 2015; Ahmed and Meehan 2016; U.S. EPA 2016a) and involve (1) the identification of possible production sites (exploration); (2) site selection and construction of a drilling place; (3) drilling, casing and cementing; (4) hydraulic stimulation; (5) production; (6) dismantling of the drilling place and renaturation. Application of frac fluids requires the following processes: (i) removing large volumes of ground- or surface water for the production of frac fluids—between 3 and 50 million L of water are pumped into each individual well (Vengosh et al. 2014; McLaughlin et al. 2016); (2) production of frac fluid, i.e., proppants and frac fluid additives are stored and mixed at the drilling site; (3) injection of frac fluids into the borehole, (4) storage and processing of the produced water; (5) disposal of the flow-back from the drilling site and produced water.

## Function and composition of frac fluids

Frac fluids induce small cracks in the relevant rock targets and allow the transport of proppants into the cracks. Major components of frac fluids are the basic fluid, additives and proppants. Proppants mostly consist of unprocessed, specified quartz sand but high-strength ceramic, sintered bauxite or zirconium oxide may also be used (Barati and Liang 2014; U.S. EPA 2016a). The function of the frac fluid additives is to increase the viscosity of the fluid, and to reduce corrosion of the bore and microbial growth. Frac fluids can be water or water/gel based. Water-based systems dominate in current HF, while alternatives constitute only ~ 2% (U.S. EPA 2015). Water-based frac fluids (slickwater fluids) contain polymers to reduce friction and are used in reservoirs with low permeability, such as clay. Slickwater frac fluids are currently mostly used for the extraction of shale gas (Gandossi and von Estorff 2015). They have a lower viscosity than gel fluids and transport proppants less efficiently into the cracks; therefore, larger volumes of water and higher pressure are required. By contrast, gel fluids are used in the formation with a higher permeability. Despite the higher water consumption of slickwater fluids, they are more cost-effective, easier to produce and offer the possibility of water recycling.

Alternative frac fluids consist of foamed materials or emulsions that are generated by the use of nitrogen, carbon dioxide, hydrocarbons and methanol (Montgomery 2013; Saba et al. 2012; Gupta and Hlidek 2009; Gupta and Valkó 2007). Moreover, acid-based frac fluids are used for HF in carbonate formations without the addition of proppants. A particular challenge for HF is in rock formations where the injection of water reduces permeability. Here, non-water-based fluids are used that consist of petroleum distillates and propane and, usually, further additives. The use of non-water-based frac fluids has decreased in recent years due to the improvement of safety and health considerations and water-based techniques. Nevertheless, the use of hydrocarbons such as diesel or kerosene is still allowed according to the revised criteria of the U.S. EPA (2014). Therefore, typical compound groups in frac fluids are gelling agents, thickening agents, stabilizers of clay, biocides, solubilizers, viscosity modifiers, surface tension reducers, buffers, and anti-foam agents (Stringfellow et al. 2014, 2017a; Elsner and Hoelzer 2016; U.S. EPA 2016a; King and Durham 2015; Kahrilas et al. 2015). There are more than 1100 chemicals listed as potentially present in frac fluids (U.S. EPA 2011). The Tyndall Centre Manchester (2011) provides an overview of 260 additives, 750 chemicals and additional components that have been used in 2500 different frac fluids between 2005 and 2009. Chemicals used for HF in the USA are listed in FracFocus (http://fracfocus.org), which is organized by the US Groundwater Protection Council and the Interstate Oil and Gas Compact Commission (IOGCC). The British Columbia Oil and Gas Commission provides an analog platform (http://fracfocus.ca/en) in Canada. Indeed, compounds in frac fluids are increasingly made public (International Association of Oil and Gas Producers (IOGP) 2017; FracFocus 3.0 2020; Cuadrilla 2017). Chemicals used in Germany are listed on the website of the ‘Bundesverband Erdgas, Erdöl und Geoenergie e. V.’ (BVEG 2017). A comprehensive list of frac additives is also available in Meiners et al. (2012a, b). An overview of frequently used frac fluid chemicals and their function in the fluid is given in Table 1.

**Table 1:** Frac fluid additives, their function in the fluid and corresponding chemicals (U.S. EPA 2016a, modified)

Additive	Function	Chemicals reported in 20% or more of disclosures in the EPA FracFocus 1.0 project database for given additive (U.S. EPA 2015)^a^
(Inorganic) Acid	Dissolves cement, minerals, and clays to reduce clogging of the pore space	Hydrochloric acid
Biocide	Controls or eliminates bacterial growth, which can be present in the base fluid and may have detrimental effects on the long-term well productivity	Glutaraldehyde; 2,2-dibromo-3-nitrilopropionamide
Breaker	Reduces the designed increase in viscosity of specialized treatment fluids such as gels and foams after the proppant has been placed and flow-back commences to clean up the well	Peroxydisulfuric acid diammonium salt
Clay control	Prevents the swelling and migration of formation clays that otherwise react to water-based fluids	Choline chloride
Corrosion inhibitor	Protects the iron and steel components in the wellbore and treating equipment from corrosive fluids	Methanol; propargyl alcohol; isopropanol
Crosslinker	Increases the viscosity of base gel fluids by connecting polymer molecules	Ethylene glycol; potassium hydroxide; sodium hydroxide
Emulsifier	Facilitates the dispersion of one immiscible fluid into another by reducing the interfacial tension between the two liquids to achieve stability	Polyoxyethylene(10)nonylphenyl ether; methanol; nonyl phenol ethoxylate
Foaming agent	Generates and stabilizes foam fracturing fluids	2-Butoxyethanol; nitrogen, liquid; isopropanol; methanol; ethanol
Friction reducer	Reduces the friction pressures experienced when pumping fluids through tools and tubulars in the wellbore	Hydrotreated light petroleum distillates
Gelling agent	Increases fracturing fluid viscosity allowing the fluid to carry more proppant into the fractures and to reduce fluid loss to the reservoir	Guar gum; hydrotreated light petroleum distillates
Iron control agent	Controls the precipitation of iron compounds (e.g., Fe_2_O_3_) from solution	Citric acid
Nonemulsifier	Separates problematic emulsions generated within the formation	Methanol; isopropanol; nonyl phenol ethoxylate
pH control	Regulates the pH of a solution by either inducing a change (pH adjuster) or stabilizing and resisting change (buffer) to achieve desired qualities	Carbonic acid, dipotassium salt; potassium hydroxide; sodium hydroxide; acetic acid
Resin curing agents	Lowers the curable resin-coated proppant activation temperature when bottom hole temperatures are too low to thermally activate bonding	Methanol; nonyl phenol ethoxylate; isopropanol; alcohols, C12–14-secondary, ethoxylated
Scale inhibitor	Controls or prevents scale deposition in the production conduit or completion system	Ethylene glycol; methanol
Solvent	Controls the wettability of contact surfaces or prevents or breaks emulsions	Hydrochloric acid

^a^This compilation considers 32,885 frac fluid recipes including 615,436 individual components

A similar compilation has been published, comprising 35 chemicals present in at least 10% of frac fluids (U. S. EPA 2016a). Besides chemicals listed in Table 1, this compilation includes the following substances and compound groups: quartz, sodium chloride, mineral oil, naphthalene, 2,2-dibromo-3-nitrilopropionamide, phenolic resins, hexamethylenetetramine, 1,2,4-trimethylbenzene, benzalkonium chloride, 4-nonylphenol (branched, ethoxylated, polymer), formic acid, sodium chlorite, tetrakis (hydroxymethyl), phosphonium sulfate (2:1), polyethylene glycol, ammonium chloride and sodium persulfate. Methanol represents the most frequently mentioned chemical, present in approximately 72% of all frac fluids.

Some chemicals are added to frac fluids as tracers to control the efficiency of injection into rock formations (U.S. EPA 2016a) and to detect possible contaminations of the environment (Kurose 2014). These tracers include thiocyanates, fluorobenzoic acids, alkyl esters, and radioactive tracers such as titrated water or methanol. Due to the multitude of applied chemicals and different requirements depending on the specific geological conditions, a general recipe of all frac fluids is not available. The composition of frac fluids is highly variable. Furthermore, the application of chemicals changes rapidly since companies are constantly optimizing the processes. A trend is to use fewer and less hazardous chemicals (Gandossi and von Estorff 2015; Wang and Fan 2015; Kassner 2016; Halliburton 2017; Schlumberger 2017). The use of nanomaterials in frac fluids is still at a more basic level of research and development (Gottardo et al. 2016). While publication of the components of frac fluids in registers such as FracFocus 3.0 was previously performed on a voluntary basis, it is a legally binding duty in Germany since 2016.

Chemical transformation processes of frac fluid components are not well investigated and only scarce information is available. It is likely that certain chemicals, e.g., oxidants, undergo chemical reactions in the subsurface considering the high temperatures of 50–100 °C at depths of 1000–2500 m, the high pressure and high salinity (Hoelzer et al. 2016). These chemical reaction products can be expected to appear in flow-back and produced water; however, their identity has not yet been systematically studied.

## Consumption of water and frac fluids

Consumption of 8000–100,000 m^3^ water per unconventional well have been reported for six shale gas plays in the period between 2000 and 2011 (Vengosh et al. 2014). A representative study for major shale gas plays in Texas estimated the water consumption of 14,900 horizontal drilling operations in the Barnett shale, 390 in the Haynesville Formation and 1040 in the Eagle Ford Formation (Nicot and Scanlon 2012). During the 2009-6/2011 period, median water use per horizontal well was 10,600 m^3^, 21,500 m^3^, and 16,100 m^3^ in the three areas, respectively. In another study, by fitting a normal distribution to freshwater withdrawal volumes, an average water consumption was estimated to be 15,000 m^3^ per single well in the Marcellus Shale Formation in Pennsylvania. Based on well completion reports submitted to the Pennsylvania Department of Environmental Protection (PADEP) in 2010, it is indicated that 3500–26,000 m^3^ is required to hydraulically fracture a single well (Jiang et al. 2014). Using published scientific literature data from 2010 to 2014, water demand of 8000–19,000 m^3^ per well lifespan was estimated for a Polish shale gas production site (Vandecasteele et al. 2015). Data from Germany range from 37 to 4040 m^3^ water/frac and consumption of additives of 615–274,764 kg/frac (Meiners et al. 2012a, b). On the basis of modeling future HF operations in the USA in two scenarios of drilling rates, Kondash et al. (2018) projected cumulative water use and flow-back/produced water volumes to increase by up to 50-fold in unconventional gas-producing regions and up to 20-fold in unconventional oil-producing regions between 2018 and 2030, assuming that the growth of water use matches current growth rates of HF production.

A relatively new aspect is to reuse the flow-back/production water. After high-pressure pumping of frac fluids into rock formations, the injected fluid returns to the surface via the borehole. Initially, the fraction of frac fluid is higher compared to formation water, the natural layer of water inside gas or oil reservoirs. Later, the fraction of frac fluid declines (NYSDEC 2011) and the production water consists predominantly of formation water. In principle, production water can be recycled to reduce the need for freshwater and chemicals (Leiming et al. 2016). Despite the advantages of this method, according to data from ten states in the USA (U.S. EPA 2016a), the fraction of recycled frac fluids by reusing production water is only about 5%.

## Toxicity of frac fluids

The public discussion on HF has focused predominantly on the hazardous substances present in frac fluids. In the EU, the classification of the applied chemicals is performed according to European chemicals legislation (Gottardo et al. 2013; COM 2014). The CLP Regulation (classification, labeling and packaging of substances and mixtures) (Regulation (EC) No. 272/2008) is the basis of classification and labeling for the required technical dossiers; it comprises ten health hazard classes (Table 2) and 16 physio-chemical hazard classes, as well as a class for environmental hazards.

**Table 2:** Classification of health hazards (Regulation 1272/2008/EC, Part 3 of Annex I)

Hazard class	Hazard class
Acute toxicity	Carcinogenicity
Skin corrosion/irritation	Reproductive toxicity
Serious eye damage/eye irritation	Specific target organ toxicity—single exposure
Respiratory or skin sensitization	Specific target organ toxicity—repeated exposure
Germ cell mutagenicity	Aspiration hazard

Two questions are particularly relevant when considering the hazard of chemicals used for HF: (a) What are the hazard characteristics of the individual compounds? (b) Do the applied frac fluid mixtures belong to the categories ‘hazardous for human health’ or ‘hazardous to the environment’ according to chemicals legislation? With respect to (a), frac fluids have been shown to contain hazardous compounds according to the GHS/CLP. These regulations are binding concerning transport, storage and use of the chemicals. Several comprehensive reviews are available which provide an overview of the classification of chemicals in frac fluids (Meiners et al. 2012a, b; Stringfellow et al. 2014; Elsner and Hoelzer 2016; Xu et al. 2019). Several compounds in frac fluids, such as biocides, have been classified as hazardous and are also used in consumer products. A well-known example is the biocide Kathon CG (CAS RN 55965-84-9), a mixture of 5-chloro-2-methyl-2H-isothiazole-3-on (C(M)IT) and 2-methyl-2H-isothiazole-3-on (MIT) at a ratio of 3:1. C(M)IT/MIT (3:1) has been approved by Commission Implementing Regulation (EU) 2016/131 of 1 February 2016 (COM 2016) to be used, e.g., in private area and public health area disinfectants and other biocidal products, and food and feed area disinfectants. In 2018, C(M)IT/MIT has been classified more strictly as hazard category 2 regarding acute toxicity after inhalation and dermal exposure. Furthermore, the issue of skin and eye irritation has also been comprehensively addressed (COM 2018) (Table 3).

**Table 3:** Classification of reaction mass of 5-chloro-2-methyl-2H-isothiazol-3-one and 2-methyl-2H-isothiazol-3-one (3:1) and 2-methylisothiazol-3(2H)-one

Chemical name	Hazard class and category code(s)	Hazard statement code(s)
Reaction mass of 5-chloro-2-methyl-2H-isothiazol-3-one and 2-methyl-2H-isothiazol-3-one (3:1)^a^	Acute Tox. 3	H301 (toxic if swallowed)
	Acute Tox. 2	H310 (fatal in contact with skin)
	Acute Tox. 2	H330 (fatal if inhaled)
	Skin Corr. 1C	H314 (causes severe skin burns and eye damage)
	Skin Sens. 1A	H317 (may cause an allergic skin reaction)
	Skin Irrit. 2	H315 (causes skin irritation)
	Eye Irrit. 2	H319 (causes serious eye irritation)
	Aquatic Acute 1	H400 (very toxic to aquatic life)
	Aquatic chronic 1	H410 (very toxic to aquatic life with long-lasting effects)
2-Methylisothiazol-3(2H)-one^a^	Acute Tox. 2	H330 (fatal if inhaled)
	Acute Tox. 3	H311 (toxic in contact with skin)
	Acute Tox. 3	H301 (toxic if swallowed)
	Skin Corr. 1B	H314 (causes severe skin burns and eye damage)
	Eye Dam. 1	H318 (causes serious eye damage)
	Skin Sens. 1A	H317 (may cause an allergic skin reaction)
	Aquatic Acute 1	H400 (very toxic to aquatic life)
	Aquatic Chronic 1	H410 (very toxic to aquatic life with long-lasting effects)

Entry in Annex VI, Regulation CLP

^a^13th Adaptation to Technical Progress (ATP) (COMMISSION REGULATION (EU) 2018/1480 of 4 October 2018)

Classification of compounds frequently used in frac fluids according to CLP is given in Table 4. The listed chemicals were present in at least 20% of all frac fluids listed in the U.S. EPA FracFocus 1.0 project database (U.S. EPA 2015, 2016a, Appendix C, Table C-2). This classification only informs about the intrinsic toxicity (hazard) of the compounds; however, conclusions with respect to health risks require additional information about exposure scenarios. Elements of a hazard-based approach in legal requirements of hydrofracking can be found in German water law: frac fluid mixtures are only permitted if they are classified as ‘not hazardous to water’ or ‘low hazardous to water’.

**Table 4:** Classification of commonly used frac chemicals (according to U.S. EPA 2016a) in accordance with the requirements of the CLP Regulation

International chemical identification	CAS RN	Classification	
		Hazard class and category code(s)	Hazard statement code(s)
2,2-Dibromo-3-nitrilopropionamide^a^	10,222-01-2		
2-Butoxyethanol	111-76-2	Acute Tox. 4 Acute Tox. 4 Acute Tox. 4 Eye Irrit. 2 Skin Irrit. 2	H332 H312 H302 H319 H315
Prop-2-yn-1-ol; propargyl alcohol	107-19-7	Flam. Liq. 3 Acute Tox. 3 Acute Tox. 3 Acute Tox. 3 Skin Corr. 1B Aquatic Chronic 2	H226 H331 H311 H301 H314 H411
Diammonium peroxodisulfate; ammonium persulfate	7727-54-0	Ox. Sol. 3 Acute Tox. 4 Eye Irrit. 2 STOT SE 3 Skin Irrit. 2 Resp. Sens. 1 Skin Sens. 1	H272 H302 H319 H335 H315 H334 H317
Choline chloride^b^	67-48-1		
Acetic acid	64-19-7	Flam. Liq. 3 Skin Corr. 1A	H226 H314
Ethanol; ethyl alcohol	64-17-5	Flam. Liq. 2	H225
Alcohols, C12–14-secondary, ethoxylated^b^	84,133-50-6		
Ethanediol; ethylene glycol	107-21-1	Acute Tox. 4	H302
Liquid nitrogen^c^	7727-37-9		
Glutaral; glutaraldehyde; 1,5-pentanedial	111-30-8	Acute Tox. 3 Acute Tox. 3 Skin Corr. 1B Resp. Sens. 1 Skin Sens. 1 Aquatic Acute 1	H331 H301 H314 H334 H317 H400
Guar gum, propoxylated^b^	39,421-75-5		
Distillates (petroleum), hydrotreated light; kerosine—unspecified;	64,742-47-8	Asp. Tox. 1	H304
Propan-2-ol; isopropyl alcohol; Isopropanol	67-63-0	Flam. Liq. 2 Eye Irrit. 2 STOT SE 3	H225 H319 H336
Potassium carbonate^b^	584-08-7, 6381-79-9 (sesquihydrate		
Potassium hydroxide; caustic potash	1310-58-3	Acute Tox. 4 Skin Corr. 1A	H302 H314
Methanol	67-56-1	Flam. Liq. 2 Acute Tox. 3 Acute Tox. 3 Acute Tox. 3 STOT SE 1	H225 H331 H311 H301 H370
Sodium hydroxide; caustic soda	1310-73-2	Skin Corr. 1A	H314
Polyethylene glycol nonylphenyl ether^d^	9016-45-9		
Hydrochloric acid	231-595-7	Skin Corr. 1B STOT SE 3	H314 H335
Citric acid^b^	77-92-9		

The listed chemicals were present in at least 20% of all frac fluids listed in the U.S. EPA FracFocus 1.0 project database (U.S. EPA 2016a, Appendix C, Table C-2)

^a^In: LIST OF PENDING ARTICLE 95(1) APPLICATIONS. Prepared as of 15 December 2015. ^b^Notified classification and labeling according to CLP criteria. ^c^Not classified. ^d^Committee for Risk Assessment (RAC) Opinion on an Annex XV dossier proposing restrictions on Nonylphenol and Nonylphenol ethoxylates: “NONYLPHENOL AND NONYLPHENOLETHOXYLATES IN TEXTILES”

A general problem which can arise during risk evaluation of frac fluids is given by the sometimes imprecise or missing description of their chemical composition and the chemical identity of individual compounds (Elsner et al. 2015) and/or the lack of toxicological data for the classification of individual frac chemicals as well as frac fluid mixtures. Compared to the information available for individual frac chemicals, only little is known about complete frac fluid mixtures. Exceptions are self-classifications by users (e.g., ExxonMobile 2017). Risk assessment of frac fluids should refer to the total (finally applied) mixture, including the basic fluid, specific additives and proppants; this mixture should be evaluated based on the ratio of individual compounds of the entire volume (Regulation (EC) No 1272/2008 (CLP), article 2, 2008). In contrast to this regulation, some authors assessed only specific mixtures of additives and not the complete frac fluid. Using this procedure, Meiners et al. (2012a, b) concluded that six of 88 analyzed additive mixtures should be classified as toxic, six as dangerous to the environment, 25 as harmful to human health, 14 as irritating, 12 as corrosive, and 27 as non-hazardous. However, in the finally used (complete) frac fluids, the concentrations of these compounds or mixtures of additives are so low that thresholds of the Regulation (EC) no. 1272/2008 (CLP) (2008) are usually not exceeded. Therefore, in many cases, the complete frac fluid mixtures can be classified as non-hazardous to human health (Ewers et al. 2013; Gordalla et al. 2013). Self-classifications of ExxonMobile (2017) came to the conclusion that the total fluid is weakly hazardous to water and not hazardous to the environment. According to this assessment, labeling of the considered frac fluids would not be necessary (ExxonMobile 2017).

**Fig. 2:**
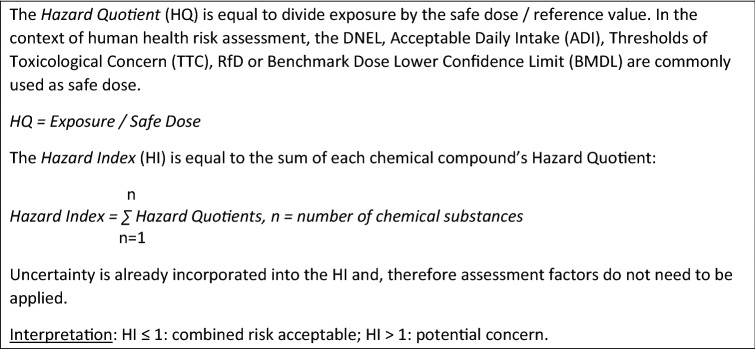
The Hazard Index approach to assess combined exposures to multiple chemicals (OECD 2018; NRC 2009)

Another approach for the assessment of risks for health and ecological impacts by constituents of the frac fluid was introduced by Bergmann et al. (2014). The authors defined a *risk quotient* by dividing the substance’s concentration in the frac fluid by an assessment value. The assessment values correspond to threshold values for groundwater (LAWA 2017), guidance values for drinking water, or precautionary values of drinking water for substances that cannot (or can only partially) be toxicologically assessed (Dieter 2014). If a compound has a risk quotient < 1, no risk can be expected, while a risk quotient ≥ 1 suggests a possibility of increased risk. A high risk can be expected if the risk quotient exceeds a value 1000. Using this approach, the authors concluded that six of eight evaluated substances used in frac fluids lead to a high risk to human health. OECD (2018) and NRC (2009) describe a similar approach with the metrics *hazard quotient* (for an individual compound) and *hazard index* when reviewing the assessment of combined exposures (Fig. 2). It should, however, be kept in mind that the approach by Bergmann et al. (2014) uses the principles of drinking water assessment and, as such, may be critically discussed whether it represents an adequate basis. For example, the threshold values for groundwater were often justified by drinking water limit values or comparable derived toxicological guidance values. Toxicological based regulations for drinking water are based on the principle that 2 L of water per day can be consumed throughout life without an increased risk to human health. Although high standards should also be maintained concerning frac fluids, the intended use differs widely from that of drinking water and may, therefore, require different procedures for risk assessment.

A multi-criteria decision analysis (MCDA) framework to estimate potential risks for drinking water resources by frac chemicals was presented by Yost et al. (2017). The MCDA is based on their toxic hazard [chronic Reference Doses (RfDs) and Oral Slope Factors for non-cancer and cancer endpoints], the frequency of use, physio-chemical properties, and their mobility in water. It allows a nationwide or state-specific analysis ranking of frac fluid components. The nationwide analysis (U.S.) of the non-cancer MCDA (37 chemicals in total) indicates the highest Total Hazard Potential Scores for propargyl alcohol, 2-butoxyethanol, *N*,*N*-dimethylformamide, acrylamide, and naphthalene (ranked from high to low). For 2-butoxyethanol, *N*,*N*-dimethylformamide, and naphthalene, the *Occurrence Score* and the *Physical Properties Score* shape the ranking. The cancer MCDA, based on the nationwide analysis of ten chemicals in total, shows acrylamide, bis(2-chloroethyl) ether, quinoline, 1,4-dioxane, and benzyl chloride with the highest Total Hazard Potential Scores. For the first three substances, the tox score determines the overall score.

## Produced water

‘Produced water’ (or ‘production water’) has been defined as any type of water that flows to the surface from oil and gas wells (U.S. EPA 2016a). It represents a mixture of flow-back (i.e., injected fluid returning to the surface) and reservoir water, i.e., the water present in natural oil and gas deposits. Early after hydraulic stimulation, e.g., within 1 or 2 days, the produced water contains a relatively high fraction of flow-back with frac fluids. Later, the fraction of reservoir water in the produced water increases. Produced water contains a complex mixture of potentially harmful inorganic and organic chemicals from naturally occurring geogenic compounds, constituents of the frac fluid, and transformation products from biotic and abiotic processes [Hoelzer et al. 2016; Sun et al. 2019 (review)]. Substantial constituents in produced water are the following chemical groups:

- inorganic salts including those from chloride, bromide, sulfate, sodium, magnesium and calcium;
- metals including barium, manganese, iron, and strontium;
- radioactive materials including radium-226 and radium-228;
- oil, grease and dissolved organics, including BTEX;
- hydraulic fracturing chemicals, including tracers and their transformation products;
- produced water treatment chemicals.

Interacting factors that can influence the chemical composition of produced water include the composition of injected hydraulic fracturing fluids; the targeted geological formation and associated hydrocarbon products; the stratigraphic environment; subsurface processes and the residence time. Therefore, very different types of contamination have been observed in produced waters. Already in flow-back and produced water, more than a thousand geogenic organic compounds have been identified using GC-FID, GC–MS, and GC × GC–TOF–MS techniques but only in a qualitatively manner (Luek and Gonsior 2017). Tables 5 and 6 summarize organics, inorganics and further parameters analyzed in produced water that have been reported quantitatively above the limit of detection. The use of U.S. EPA’s drinking water MCLs (Maximum Contaminant Levels) to assess the toxicological risk of chemicals in produced water, as it has been performed by some authors (Akob et al. 2015; Ziemkiewicz and He 2015; Sun et al. 2019), appears inadequate because produced water on its own is not a subject of protection.

**Table 5:** Concentrations of organic parameters in produced water from unconventional reservoirs (including shale, tight formation, and coalbed methane)

Parameters	Shale formation			Tight formation	Coalbed methane			
	Barnett	Marcellus		Cotton Valley Group	Powder river	Raton	San Juan	Black Warrior
TOC (mg/L)	*9.75* (6.2–36.2)	160 (1.2–1530)	*89.2* (1.2–5680)	198 (184–212)	3.52 (2.07–6.57)	1.74 (0.25–13.00)	2.91 (0.95–9.36)	6.03 (0.00–103.00)
DOC (mg/L)	*11.2* (5.5–65.3)		*117* (3.3–5960)		3.18 (1.09–8.04)	1.26 (0.30–8.54)	3.21 (0.89–11.41)	3.37 (0.53–61.41)
BOD (mg/L)	*582* (101–2120)		*141* (2.8–12,400)					
Benzene (µg/L)	680 (49–5300)		*220* (5.8–2000)			4.7 (BDL–220.0)	149.7 (BDL–500.0)	
Toluene (µg/L)	760 (79–8100)		*540* (5.1–6200)			4.7 (BDL–78.0)	1.7 (BDL–6.2)	
Ethylbenzene (μg/L)	29 (2.2–670)		*42* (7.6–650)			0.8 (BDL–18.0)	10.5 (BDL–24.0)	
Xylenes (μg/L)	360 (43–1400)		*300* (15–6500)			9.9 (BDL–190.0)	121.2 (BDL–327.0)	

The data are given as average (min.–max.) or *median* (min.-max.) (from U.S. EPA 2016a, Appendix E; modified)

The data sources corresponding to U.S. EPA (2016a) (Appendix E, Table E-9, modified) were Hayes and Severin (2012), Barbot et al. (2013), Hayes (2009), Blondes et al. (2014), Dahm et al. (2011), and DOE (2014)

**Table 6:** Levels of inorganic and organic parameters in flow-back and produced water from unconventional reservoirs

Authors (year)	Parameters	Concentration	Matrix, study site
Akob et al. (2015)	Barium	15,000 mg/L (median)	Produced water
		3780–22,400 mg/L (range)	Marcellus shale, Burket shale
	Chloride	109,000 mg/L–184,000 mg/L	(Pennsylvania)
	Sodium	44,800 mg/L–63,100 mg/L	
	Calcium	16,300–39,200 mg/L	
	Strontium	3390–10,300 mg/L	
	Bromide	760–1470 mg/L	
	Non-volatile dissolved	6.7–49.3 mg/L	
	Organic carbon (NVDOC)		
	Low molecular-weight	0.7–5.6 µg/L	
	Organic acid anion (LMWOA)		
	Benzene	< 1.0–1,8 µg/L	
	Toluene	1.0–1.3 µg/L	
	Tetrachloroethylene	< 1.0–11.7 µg/L	
Lester et al. (2015)	Aluminum	0.064 mg/L	Flow-back; Denver–
	Arsenic	0.067 mg/L	Julesburg (Colorado)
	Boron	3.105 mg/L	
	Barium	8.542 mg/L	
	Calcium	524.1 mg/L	
	Chromium	0.058 mg/L	
	Cesium	0.073 mg/L	
	Copper	0.288 mg/L	
	Iron	81.42 mg/L	
	Potassium	101.3 mg/L	
	Lithium	3.519 mg/L	
	Magnesium	106.4 mg/L	
	Manganese	1.471 mg/L	
	Sodium	6943.9 mg/L	
	Nickel	0.042 mg/L	
	Rubidium	0.230 mg/L	
	Silicon	19.65 mg/L	
	Strontium	60.25 mg/L	
	Titanium	0.028 mg/L	
	Vanadium	0.120 mg/L	
	Zinc	0.051 mg/L	
	Acetone	16,000 µg/L	
	2-Butanone	240 µg/L	
	Xylenes	30 µg/L	
	1,4-Dioxane	60 µg/L	
	2-Methylphenol	150 µg/L	
	3- and 4-Methylphenol	170 µg/L	
	2-Methylnaphthalene	4 µg/L	
	Dimethyl phthalate	15 µg/L	
	Phenanthrene	3 µg/L	
	Pyrene	0.9 µg/L	
	Butyl benzyl phthalate	4.2 µg/L	
	Bis(2-ethylhexyl) phthalate	29 µg/L	
	Phenol	830 µg/L	
	2,4-Dimethylphenol	790 µg/L	
Ziemkiewicz and He (2015)	Barium	10.2 mg/L; 2580 mg/L; 514.68 mg/L (minimum, maximum, mean)	Flow-back,
	Strontium	117 mg/L; 4660 mg/L; 1365 mg/L	Marcellus Shale
	Natrium	2440 mg/L; 119,000 mg/L; 26,202 mg/L	(West Virginia)
	Magnesium	107 mg/L; 2260 mg/L; 835 mg/L	
	Calcium	1010 mg/L; 19,900 mg/L; 7269 mg/L	
	Potassium	44.2 mg/L; 488 mg/L; 260.66 mg/L	
	Iron	14.7 mg/L; 149 mg/L; 67.08 mg/L	
	Manganese	1.38 mg/L; 10.2 mg/L; 5.5 mg/L	
	Arsenic	Nd	
	Chromium	Nd; 0.14 mg/L; 0.085.5 mg/L	
	Mercury	Nd	
	Lead	Nd; 0.1 mg/L; 0.1 mg/L	
	Selenium	Nd; 0.34 mg/L; 0.26 mg/L	
	Silver	Nd	
	Aluminium	Nd; 13.3 mg/L; 4.61 mg/L	
	Zinc	Nd; 0.35 mg/L; 0.14 mg/L	
	Nitrate	Nd; 0.3 mg/L; 0.02 mg/L	
	Nitrite	Nd; 0.8 mg/L; 0.06 mg/L	
	Sulfate	Nd; 108 mg/L; 55.93 mg/L	
	Chloride	4700 mg/L; 79,000 mg/L; 42,683 mg/L	
	Phosphate	Nd; 90 mg/L; 9.49 mg/L	
	Bromide	52.5 mg/L; 970 mg/L; 465.96 mg/L	
	Benzene	Nd; 372 µg/L; 194.47 µg/L	
	Ethylbenzene	Nd; 235 µg/L; 85.34 µg/L	
	Styrene	Nd; 141 µg/L; 141 µg/L	
	Toluene	Nd; 2450 µg/L; 621.71 µg/L	
	Xylene (*m*,*p*)	Nd; 3380 µg/L; 825.75 µg/L	
	Xylene (*o*)	Nd; 673 µg/L; 205.5 µg/L	
	MBAS	Nd; 0.61 mg/L; 0.42 mg/L	
	Gross Alpha	1.84 pCi; 20,920 pCi; 5866 pCi	
	Gross Beta	9.6 pCi; 4664 pCi; 1172 pCi	
	Radium-226	15.4 pCi;1194 pCi; 358 pCi	
	Radium-228	4.99 pCi; 216 pCi; 94.6 pCi	
	Thorium-228	0.3 pCi; 2.35; 1.29 pCi	
	Thorium-230	0 pCi; 9.37 pCi; 2.13 pCi	
	Thorium-232	0 pCi; 0.38 pCi; 0.07 pCi	
	Uranium-238	n/a; n/a; 0.34 pCi	
	Potassium-40	Nd 221 pCi; 62.44 pCi	
Ziemkiewicz (2013)	Benzene	6 µg/L; 19.7 µg/L; 21 µg/L (flow-back cycle at days 7, 14 and 35)	Flow-back, dry well
	Toluene	3.8 µg/L; 12 µg/L; 6.8 µg/L	Marcellus Shale
	Xylene (*m*,*p*)	0.7 µg/L; 6.2 µg/L; 3.2 µg/L	(West Virginia)
	Benzene	370 µg/L; 18 µg/L; 122 µg/L (flow-back cycle at days 7, 14 and 35)	Flow-back, wet well
	Toluene	2070 µg/L; 170 µg/L; 525 µg/L	Marcellus Shale
	Xylene (*m*,*p*)	2424 µg/L; 375 µg/L; 525 µg/L	(West Virginia)

## Environmental pollution and toxicological risks

Incidents in the surface installations of HF plants may lead to contamination of near-surface groundwater and of surface waters with frac chemicals and production water. Leaks of the drilling holes will cause contamination of the surrounding rock and groundwater. Surface waters can be contaminated by the release of insufficiently treated production water and by leakage from aboveground reservoirs for storage of production water. Recently, four mechanisms have been reported to be particularly relevant for the quality of water resources (Vengosh et al. 2014): (a) contamination of near-surface groundwater by leaking gas wells, diffusive emissions (stray gas), frac fluids and flow-back water; (b) contamination of surface water from inadequately treated production water; (c) accumulation of toxic and radioactive compounds in sediments of rivers and lakes exposed to production water or frac fluids and (d) overexploitation of water resources.

## Contamination of groundwater

The potential to contaminate groundwater has been considered to be the most relevant risk of HF (Gagnon et al. 2016; Vengosh et al. 2014). Groundwater contaminations may be caused by

- Blowout, i.e., the accidental release of flow-back, production water and hydrocarbons; release of frac fluid by leakage of containers; leakage of production water pipelines.
- Leaking boreholes by deficient casing and cementing; this also refers to leaking drainage wells for disposal of production water.
- Migration of frac fluid components from deeper into more superficial formations.
- Rising of gas (i.e., ‘thermogenic methane’).
- Rising of deposited production water from deep wells.

Frac chemicals exhibit a relatively low volatility and many of the most frequently used have a high solubility in water and a negative or very low octanol–water partition coefficient (Kow), which supports transfer into groundwater (U.S. EPA 2016a). Solubility of frac fluids may be increased by the presence of solvents, such as methanol or ethanol. Changes in common water quality parameters can be associated with impacts from hydraulic fracturing activities. Measurable changes in methane levels, total dissolved solids (TDS), ratios of geochemical constituents, and isotopic ratios might suggest an impact by HF but could also be from either natural or anthropogenic sources. Specific frac chemicals or specific tracer substances were comparatively little investigated as groundwater contaminants (U.S. EPA 2016a).

A general problem in assessing the influence of HF on the quality of groundwater represents the lack of baseline monitoring before the onset of oil or gas production. However, such baseline monitoring is an important prerequisite for a sound evaluation of possible consequences of HF, particularly in regions with former conventional oil and gas production.

A critical question is to which degree fluid may ascend from deeper formations and reach groundwater (Reagan et al. 2015). Model simulations have shown that frac fluids may ascend by only approximately 50 m even if large cracks of more than 1000 m across rock formations occur (Ewen et al. 2012). The largest possible up-flow in vertical fissure systems was estimated to be approximately 215 m under worst-case assumptions. Other authors also reported that vertical leakage over larger distances is very unlikely (Groat and Grimshaw 2012; BGR 2016). However, the horizontal flux in deep water layers in the geological setting coal seam (Münsterland, Germany) may reach several km, at a rate of ~ 20 m per year (Ewen et al. 2012).

## Methane in water due to HF activities and conventional oil and gas production

### Groundwater and drinking water

Methane in ground and drinking water is a common phenomenon already known from conventional oil production (Muehlenbachs 2011). In groundwater, it can not only be of thermogenic origin but can be formed under methanogenic conditions via a biological pathway. The discrimination between thermogenic and biogenic methane is possible by measuring the typical δ^13^C–CH_4_ and δ^2^H–CH_4_ isotope fingerprint, the ratio between methane and the sum of ethane and propane, and the percentage of helium in a water sample (McIntosh et al. 2019). In HF, methane may reach the ground and drinking water through damaged cementing and casing of boreholes (Darrah et al. 2014; Dyck and Dunn 1986; Sherwood et al. 2016). Such damage is relatively frequent, with 219 incidents concerning the integrity of a total of 6466 boreholes being reported between 2008 and 2013 (Vidic et al. 2013).

Up until now, methane has been used as an indicator substance for inadequate well integrity and geological disturbance (*stray gas*). Its use has recently been re-examined more intensively. Methane is a relatively non-toxic, colorless and odorless gas. At high concentrations of 300,000 ppm or 30% in the breathing air, it acts as an asphyxiant that displaces oxygen in the lungs and causes CNS symptoms and suffocation. Action levels for methane in air and water have been recommended not for toxicological reasons but because of the risk of explosions of air/methane mixtures (Table 7). A drinking water concentration of a similar magnitude has been proposed in Canada (3 L methane/m^3^ corresponding to 2 mg methane/L) (Ontario Government 2006). The Canadian threshold value is intended as an ‘Aesthetic Objective’. Aesthetic Objectives are established for parameters that may affect the taste, smell or color of the drinking water and are not based on thresholds of adverse health effects. Methane in drinking water causes the release of gas bubbles and violent spurting from water taps.

**Table 7:** Action level of the U.S. Department of the Interior (DOI 2001) for methane

Action level	Atmospheric (% volume)		Concentration in water (mg/L)	Soil gas (% volume)
	Occupiable spaces (homes)	Un-occupiable spaces		
Immediate action	> 1.0%	> 3.0%	> 28 mg/L	> 5.0%
Warning, investigate	> 0.5% but ≤ 1.0%	> 1.0% but ≤ 3.0%	> 10 mg/L but ≤ 28 mg/L	> 3.0% but ≤ 5.0%
Monitor to determine concentration trends	> 0.25% but ≤ 0.5%			> 1.0% but ≤ 3.0%
No immediate action	≤ 0.25%	≤ 1.0%	≤ 10 mg/L	

### Methane baseline monitoring in Lower Saxony, Germany

**Fig. 3:**
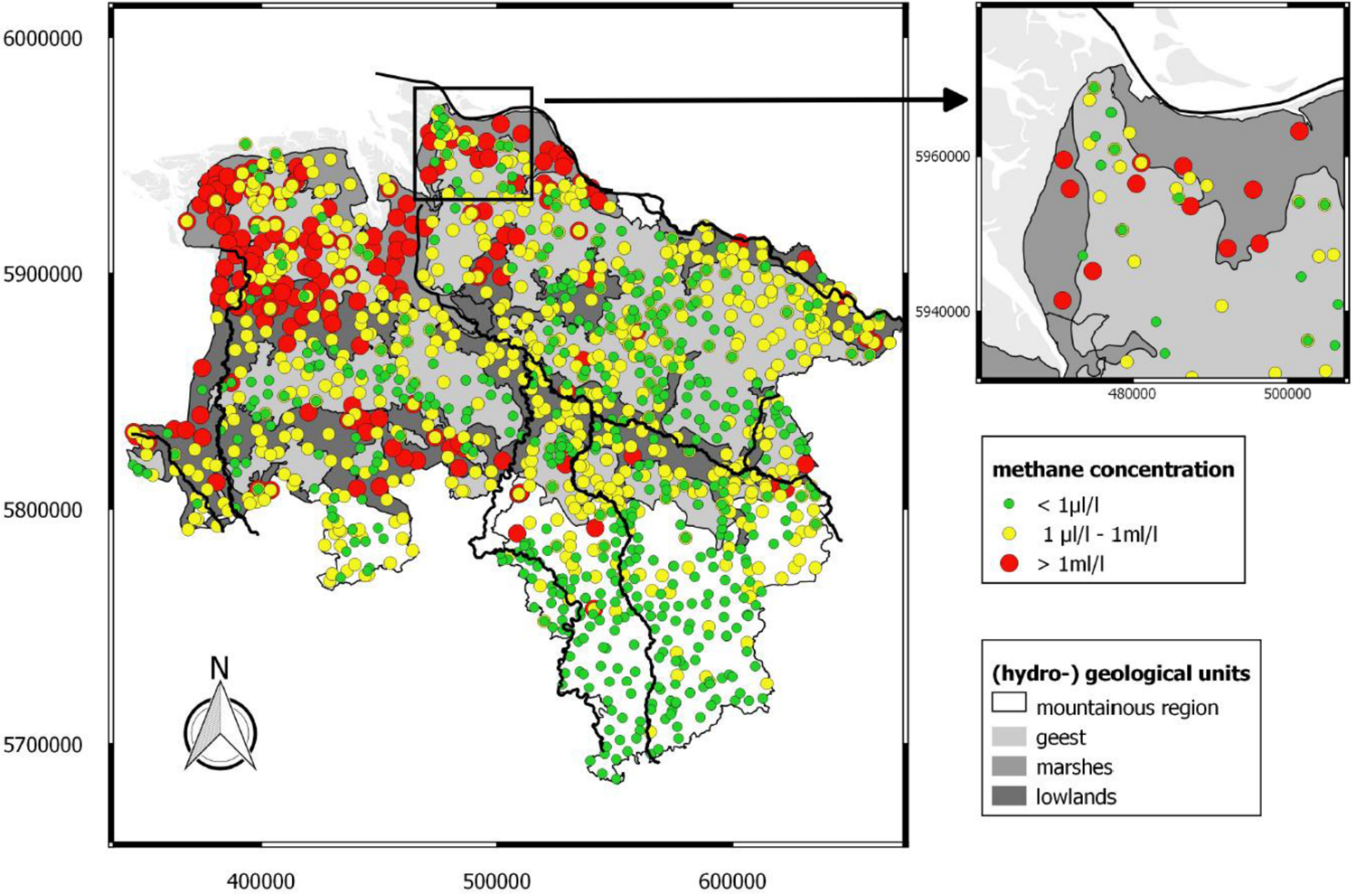
Methane in Lower Saxony’s groundwater (geodetic coordinates of sampling points according to WGS84 (1984))

It is important to monitor background levels in surface water in regions with frac activities. Therefore, a comprehensive survey has been performed in Lower Saxony (Germany), where the occurrence of methane, ethane and propane in near-surface groundwater of ~ 1000 groundwater wells was analyzed (Schloemer et al. 2018). Lower Saxony is the largest hydrocarbon province in Germany, where 327 hydraulic stimulations in 148 production wells at depths of > 3000 m have been performed since 1961 (BGR 2016). The background values for dissolved methane vary from 20 nL/L (14.2 ng/L) to 60 mL/L (42.7 mg/L) [v/v], i.e., a range of ~ 7 orders of magnitude (Fig. 3). Most analyses are indicative of methanogenic processes. Samples with high δ^13^C contents and methane levels above 10 mL/L (7.1 mg/L) can be mostly accounted for by secondary methane oxidation and biological origin, respectively. Ethane and propane were detected in 27% and 8% of all samples, with medians of 50 nL/L and 23 nL/L, respectively. Lower Saxony’s methane values indicate that 6% (*n* = 60) exceed the warning threshold of 10 mg/L and 1.3% exceed the threshold for immediate action of 28 mg/L of U.S. DOI (Table 7). The data of this survey can serve as a possible baseline tool for monitoring in future.

Several studies observed an association between the distance of gas production sites and methane in drinking water and domestic wells. Methane concentrations in drinking water wells in the Marcellus and Utica shale gas formations were 19.2 (average) and 64 (maximum) mg/L if a gas well was within a distance of 1 km (Osborn et al. 2011). These concentrations are so high that they increase the risk of fire and explosions (Table 7). By contrast, drinking water wells in the same region and a similar hydrogeology without gas wells in the neighborhood contained methane concentrations of only 1.1 mg/L (Osborn et al. 2011). Similar results were obtained in a study of 141 water wells in the Appalachian Plateau (Jackson et al. 2013). Concentrations of methane were approximately sixfold higher in drinking water wells within a distance of 1 km from gas wells compared to drinking water wells without neighboring gas production. In contrast to these findings, a third study (Siegel et al. 2015) did not observe a relationship between methane concentrations in individual home wells and oil or gas wells in the neighborhood.

## Compromised groundwater quality by vertical mixing

Mixing groundwater layers of different depths (vertical mixing) due to extensive groundwater production required for HF can compromise the quality of groundwater. One explanation for this is that high-quality groundwater is mixed with higher water layers that are contaminated by nitrates or pesticides from surface-related activities. Moreover, high-quality groundwater close to the surface can be mixed with groundwater of deeper hydrogeological formations, leading to increased concentrations of chemicals such as arsenic, chloride, fluoride, manganese, and uranium. Examples of these effects have been documented by the U.S. EPA (2016a).

## Studies of groundwater contamination by frac fluids

Studies investigating the influence of HF on groundwater quality are challenging due to potential preexisting contaminations. It has been estimated that up to three million abandoned oil and gas wells exist in the USA (Gagnon et al. 2016). This emphasizes the importance of identifying all historical data on groundwater quality in relation to previous industrial activities. DiGiulio and Jackson (2016) performed a comprehensive analysis of publicly available analytical data and reports of U.S. EPA, U.S. Geological Survey (USGS), Wyoming Oil and Gas Conservation Commission (WOGCC), and Wyoming Department of Environmental Quality (WDEQ) published from August 2009 to December 2015. They used these data to evaluate the impact on underground sources of drinking water as a result of acid stimulation and hydraulic fracturing in the Pavillion Field, Wyoming. The field comprises 181 production wells, including plugged and abandoned wells. Acid stimulation and hydraulic fracturing began in June 1960 and October 1964, respectively, and occurred only as deep as 213 and 322 m below the ground surface, respectively. These depths are comparable to deepest domestic groundwater use in the area. In response to complaints of residents regarding foul taste and odor in water from domestic wells within the Pavillion Field, the EPA conducted domestic well sampling in March 2009 and January 2010. In 2010, the U.S. EPA installed two monitoring wells (MW01 and MW02) with screened intervals at 233–239 m and 296–302 m below the ground surface, respectively. MW01 and MW02 were installed to evaluate potential upward solute transport of chemicals associated with well stimulation to maximum depths of groundwater use (~ 322 m). In general, the overall vertical groundwater gradient in the Pavillion Field is directed downwards but there is hydrological evidence of localized upwards directed hydraulic gradients, which could contribute to potential upward migration of dissolved compounds from depths of HF stimulation. Analysis of groundwater samples of MW01 and MW02 collected in 2010, 2011, and 2012 resulted in the detection of a multiplicity of frac fluids components and increased iron concentrations that indicates the pollution by HF activities. Methanol, ethanol, and isopropanol quantities of up to 863, 28.4, and 862 μg/L, respectively, were measured. Tert-butyl alcohol was detected at 6120 μg/L in one well. Tert-butyl alcohol in groundwater has been associated with degradation of tert-butyl hydroperoxide used for HF but it can also be produced by the degradation of methyl tert-butyl ether (MTBE) associated with diesel fuel. Diethylene glycol and triethylene glycol were detected in both wells at maxima of 226 and 12.7 μg/L, respectively, in MW01, and at 1570 and 310 μg/L, respectively, in MW02. Tetraethylene glycol was detected only in MW02 at 27.2 μg/L. Diesel range organics (DRO) and gasoline range organics (GRO) were detected in MW01 and MW02 with maximum DRO concentrations of 924 and 4200 μg/L, respectively, and GRO concentrations of 760 and 5290 μg/L, respectively. 1,3,5-, 1,2,4-, and 1,2,3-trimethylbenzene were measured at maximum concentrations of 71.4, 148, and 45.8 μg/L, respectively, in MW02, and at an order of magnitude lower concentrations in MW01. Naphthalene, methylnaphthalenes, and alkylbenzenes were also detected in MW02 at concentrations up to 7.9, 10.2, and 21.2 μg/L, respectively. Trimethylbenzenes and naphthalenes have been used in frac fluid mixtures. The aromatics benzene, toluene, ethylbenzene, *m*-/*p*-xylenes, and *o*-xylene were detected in MW02 at maximum concentrations of 247, 677, 101, 973, and 253 μg/L, respectively, but not in MW01. 2-Butoxyethanol, which was used extensively for well stimulation in the Pavillion Field, was detected in both monitoring wells at maxima of 12.7 μg/L. Other substances such as phenol, substituted phenols, ketones, lactate, formate, acetate, propionate, and benzoic acid were also measured quantitatively in the monitoring wells. Detection of organic compounds or degradation products of chemicals that have been used in frac fluids for production well stimulation in MW01 and MW02 provide evidence of an impact to groundwater and indicate upward solute migration to depths of groundwater use under the specific hydrogeological conditions. Additionally, the anomalous concentrations of major ions in domestic wells suggest an influence of well stimulation. Detection of DRO/GRO and further organic compounds in domestic wells < 600 m from unlined pits used up until the mid-1990s to dispose diesel–fuel-based drilling mud and production fluids suggests an impact on domestic wells. DRO and GRO in samples of these domestic wells ranged from 17.3 to 479 µg/L, and 21.6 to 48 μg/L, respectively.

A comprehensive analysis of a possible influence of shale gas production on the quality of groundwater has been performed in the Marcellus Shale, a production site in Pennsylvania (Boyer et al. 2012). A relatively high number of domestic drinking water wells (*n* = 233) in rural areas close to gas production sites were studied. Samples were taken before, as well as 8 months and up to 800 days after drilling HF activities. The authors did not observe any significant changes in drinking water after frac activities, analyzing the conventional organic and inorganic parameters of drinking water quality [e.g., pH, turbidity, TDS, dissolved organic carbon (DOC), total dissolved nitrogen (TDN), chloride, iron, barium, sodium, manganese, sulfate, magnesium, strontium, calcium, arsenic, lead, nitrate, chromium, cadmium, selenium, mercury, silver, bromide, sulfide, methane, BTEX, MBAS-tensides, oil and grease, and radioactivity]. Individual frac fluid organics were not analyzed. It should be taken into account that drinking water quality of the 233 private water wells sampled was partially already impaired before the onset of HF activities, with many values exceeding drinking water standards: pH (17% of 233 samples), TDS (3% of 233 samples), iron (20% of 222 samples), barium (1% of 218 samples), manganese (27% of 203 samples), arsenic (4% of 115 samples), turbidity (32% of 102 samples), coliform bacteria (33% of 125 samples), fecal coliform bacteria (33% of 122 samples), and lead (7% of 104 samples). Most notable was the exceedance of the drinking water standards in pre-drilling samples for both bacterial parameters by factors of more than 201, followed by manganese, iron, lead, turbidity by factors of 133, 68, 22, and 21, respectively. The BTEX aromatics were all below the limit of detection (LOD). Pre- and post-drilling methane concentrations were tested in 48 water wells. This compound was already present in about 20% of pre-drilling samples, partially at peak concentrations as high as 58.30 mg/L that led to an explicit risk of explosions. Most post-drilling methane levels were generally near or below the LOD (< 0.02 mg/L), even after drilling and frac activities had occurred. Methane increased at one drilled site to ~ 9 mg/L but this well also had a moderate level of methane before drilling occurred. The obtained data on methane concentrations from all 48 private water wells were used to compare pre- to post-drilling methane levels. Among these samples, there were no statistically significant increases in methane levels after drilling, and no statistically significant correlations to distance from drilling. Therefore, the authors interpreted these observations as a lack of impact of HF activities (Boyer et al. 2012).

Bromide is typically not detected in undisturbed groundwater and occurs in drinking water at levels well below those of health concern (WHO 2017). As bromide can be found at relatively high concentrations in formation or produced water/flow-back, it could be used as an indicator of the impact on groundwater from these sources. All pre-drilling bromide concentrations were < LOD (0.10 mg/L). However, in 1 of 26 water wells, bromide was detected at a concentration of 0.5 mg/L after well stimulation (Boyer et al. 2012). This water quality change may have been caused by mixing with existing formation water during the drilling or frac procedure. The elevated bromide concentration still falls below WHO’s (2009) health-based drinking water value of 6 mg bromide/L. None of the control water wells or wells near gas wells that had only been drilled and not fracked had measurable bromide concentrations during the post-drilling testing. The fact that the sum parameter DOC was unchanged despite the elevated bromide suggests that this parameter would not be suitable for qualitatively detecting organic frac chemicals.

A further study of drinking water quality was performed in the Barnett Shale, a production site in Texas (Fontenot et al. 2013). The authors analyzed samples from 91 private drinking water wells located at a distance of either more or less than three km from active natural gas wells, and 9 sample reference sites outside the Barnett Shale region. Some water samples from active wells within three km distance exceeded the drinking water maximum contaminant levels (MCL) of the U.S. EPA for arsenic (29 of 90 samples), selenium (2 of 10 samples), strontium (17 of 90 samples), and TDS (50 of 91 samples). The MCLs were exceeded by maximum factors of 16 (arsenic) and 2 (selenium). Samples from reference sites, as well as wells more than three km away from active natural gas wells, contained lower concentrations of arsenic, selenium, strontium and barium. However, the MCLs for TDS had already been exceeded in the historical data (1989–1999) (61% ≥ MCL) and in the non-active and reference area (78% ≥ MCL). Methanol was detectable in 29% of all samples, of which 24 samples were from active extraction area wells and ranged from 1.3 to 329 mg/L. Methanol in samples from non-active and reference area wells ranged from 1.2 to 62.9 mg/L (*n* = 5). Ethanol was detected in eight samples from active extraction area wells in concentrations ranging from 1 to 10.6 mg/L, and in four samples from non-active and reference area wells ranging from 2.3 to 11.3 mg/L. Both alcohols were often included as anticorrosive agents in frac fluids (Table 1) but can also occur naturally in groundwater and be formed as a by-product of microbial metabolism. The spatial pattern of the data suggests that elevated levels of some parameters could be attributed to different factors. These include the mobilization of geogenic components, hydrogeological changes due to a lowered groundwater line, or damaged casing/cementing. According to the authors, the evidence for a direct association of elevated concentrations in the groundwater to Barnett shale HF activities remains uncertain.

A further study analyzed water samples from 65 wells in the neighborhood of shale gas production sites in the southwest of Pennsylvania (Alawattegama et al. 2015). Here, consumer reports concerning deterioration in drinking water quality (color, taste or smell) coincided with the beginning of shale gas activities from 2009 onwards. Since 2009, 65 horizontal wells were drilled within a 4 km radius of the community, each well was stimulated on average with 13,249 m^3^ (3.5 million gal) of fluids and 1,451,496 kg (3.2 million lbs) of proppant. Initially, 57 water samples from 33 wells were collected and analyzed. Anion analysis of these water samples for chloride, bromide, fluoride, sulfate, phosphate, and nitrate indicated that none exceeded the drinking water MCLs and nitrite levels were below the LOD (0.0054 mg/L). The analysis of 31 analytes by means of ICP-MS (major ions, trace metals, inorganic chemicals, and radionuclides e.g., uranium) showed that the respective MCLs were exceeded by aluminium by a factor of 2.6 (one sample), iron by at most a factor of 1.4 (two samples), and manganese by, at most, a factor of 52.5 (25 samples). Cadmium and uranium concentrations were < 0.021 µg/L and < 0.05 µg/L, respectively. Methane was detected in 14 of the 18 wells tested, ranging from 0.33 to 1557 µg/L. These values are below the proposed methane action levels (Table 7). Ratios of methane to higher chain hydrocarbon of less than ~ 100 and δ13C–CH_4_ positive in more than 50% have been interpreted as indicative of thermogenic gas. Methane to ethane ratios in 4/5 of the 14 investigated samples were < 100. As isotopic analyses were not conducted, the origin of the measured methane remains unknown.

A very probable case of contamination of drinking water by frac fluids in the context of a near-surface to mid-depth long-reaching lateral geological perturbation has recently been published (Llewellyn et al. 2015). This study was performed because several households reported foaming of drinking water from domestic wells. Using high-resolution two-dimensional gas chromatography/mass spectrometry (GC/GC–TOF–MS), 2-butoxyethanol was identified in drinking water, which also was present in the flow-back (Llewellyn et al. 2015). Although 2-butoxyethanol was detectable only at very low concentrations of < 1 ng/L, it is very likely that it originated from frac activities. Notably, the U.S. EPA suggested this compound as an indicator of contamination due to frac chemicals (Tables 1, 4). Moreover, ethylene- and propylene glycol, as well as MBAS-tensides (methylene blue active substances), were identified in the µg/L-concentration range, which is close to the detection limit. The concentration of methane partially exceeded the action thresholds given in Table 7.

The U.S. EPA (2016a) has examined the impact of HF on drinking water resources at various individual sites. Several cases of contamination of drinking water aquifers were observed. In Killdeer, North Dakota, water quality samples were collected from three domestic wells, nine monitoring wells, two supply wells, one municipal well, and one state well from 07/2011 to 10/2012. Two study wells installed less than 20 m from the production well (NDGW08 and NDGW07) had significant differences in water quality compared to the remaining study wells. They showed differences in ion concentrations (e.g., chloride, calcium, magnesium, sodium, strontium) and tert-butyl alcohol. The lack of MTBE and other signature compounds associated with gasoline or fuels strongly suggests that a well blowout was the only source consistent with findings of high brine and tert-butyl alcohol concentrations in the two wells. The incident in Bainbridge Township, Ohio, is an example of insufficient and improperly cementing of the well. During the HF operation in 11/2007, ~ 3200 L of frac fluid flowed up the annulus and out of the well. The increasing pressure in the wellbore contributed to thr release of stray gas and resulted in the contamination of 26 private drinking water wells with methane. Another study in Mamm Creek, Colorado, demonstrated similar results. The Mamm Creek field is in an area where lost cement and shallow, gas-containing formations are common. As a consequence, methane has been found in several drinking water wells, along with seeps into local creeks and ponds. The proposed route of contamination was contaminants flowing up the well annulus and then along a fault.

The U.S. EPA reported that the most probable reason for drinking water contamination is the damage of casing and cementing of drilling holes that leads to spills (U.S. EPA 2016a). A median spill rate of 2.6 per 100 wells was reported (ranging from 0.4 to 12.2 spills per 100 wells), based on reported incidents in the three mentioned states. Not all spills reach and impact a drinking water resource. If approximately 5–20% of spills reach surface water or groundwater, a spill would be expected to occur and reach a drinking water resource at 0.05–2% of active or hydraulically fractured wells (U.S. EPA 2016a).

Leakage of chemicals of the frac fluid and of production water is considered as the most relevant cause of ground- and drinking water contamination (Costa et al. 2017). A first systematic analysis of publicly available data of leakage incidents of HF activities documents 77 cases in Colorado between July 2010 and July 2011 (Gross et al. 2013). In this period, 18,000 active wells were considered and leakages were reported for ~ 0.5% of those wells. Concentrations of benzene, toluene, ethylbenzene and xylene (BTEX) in groundwater from contaminated regions exceeded the current national Drinking Water MCLs of the U.S. EPA in 90, 30, 12 and 8% of the samples, respectively (Gross et al. 2013). Concentrations of benzene and toluene exceeded MCLs by factors of 220 and 2.2, respectively. Restorative measures led to a rapid decline of BTEX concentrations in the groundwater.

The question of whether HF leads to widespread and systematic groundwater pollution is a matter of controversy. In its draft on the “Assessment of the Potential Impacts of Hydraulic Fracturing for Oil and Gas on Drinking Water Resources” from 2015, the U.S. EPA concluded that there was no such impact, but in response to criticism of the EPA’s Science Advisory Board (U.S. EPA 2017), it made the conclusion much more open in the final report (U.S. EPA 2016a). Following this question, Hill and Ma (2017) examined whether shale gas development systematically impacts public drinking water quality in Pennsylvania. The authors used chemical analysis data limited to chemicals that are likely related to HF from 54,809 water samples spanning 5 years, beginning in 2011, for 424 groundwater-based community water systems, whose intakes lie within 10 km of at least one well pad. Water systems serve an average population of 787 (SD 1876) and have an average of 2.9 intake locations (SD 2.4). A difference-in-difference strategy was employed that compares, for a given community water system, water quality after an increase in the number of drilled well pads to background levels of water quality in the geographic area as measured by the impact of more distant well pads. Drilling an additional well pad within 1 km of groundwater intake locations increases shale gas-related contaminants by an average of 1.5–2.7%. The authors concluded from their results that the health impacts of HF through water contamination remain an open question.

A pilot study in three rural communities of Lower Saxony (Germany) indicated that there was no effect of frac operations in a tight gas reservoir on the groundwater quality of the near-surface aquifer. Since 1980, 53 frac operations have been performed in this deposit. Water from domestic wells in the neighborhood of the natural gas production was sampled during 2014 and 2015. A comprehensive analysis according to the German Drinking Water Ordinance showed no critical contaminations with BTEX, polycyclic aromatic hydrocarbons or metals, or substances from a non-target-GC–MS screening (Wollin et al. 2015a, b). Specific frac chemicals could not be detected. In addition, well water in the neighborhood of an injection well for disposal of oil- and gas-related wastewater did not show critical pollution by the disposed produced water (Wollin 2016a).

A recent study evaluated the water quality of private water wells in a county in Texas (Granados et al. 2019). Furthermore, the survey included questions regarding water quality, as well as an assessment of the individual health status of 75 residents living within the Eagle Ford Shale region. Well water samples (*n* = 19) from volunteers were tested for a variety of water quality parameters (inorganic cations and anions, sum parameters, frac fluid-related alcohols, aromatic compounds, aldehydes, amines, and chlorinated compounds). Of the private wells sampled, seven exceeded the U.S. EPA’s drinking water MCLs for chloride, nitrate, sulfate, and strontium. In one of the 19 wells, concentrations of the frac fluid-related chemicals, methanol, ethanol, and isopropyl alcohol, were 150, 20, and 90 mg/L, respectively. For methanol, for which there is no MCL available, a drinking water value of 14 mg/L can be derived on the basis of the reference dose (RfD) for methanol by the oral route of 2.0 mg/kg body weight per day (U.S. EPA IRIS 2013) and using a default allocation factor of the RfD of 20%. The analyzed methanol concentration of 150 mg/L exceeds this value by a factor of 11. From the 75 participants of the study, the main three sources of drinking water were reported to be the home city water supply (*n* = 17), private wells (*n* = 14), and grocery store/purchased water (*n* = 44). Of note, confidence in safety to drink home tap water was highest in the group of participants using private wells (13 of 14 wells). The majority of the participants did not have confidence in the quality of their drinking water, with many reporting changes in smell and appearance.

## Contamination of groundwater by activities above the surface

### Organic compounds

Numerous specific organic chemicals are used during HF activities. A comprehensive study was performed in the Marcellus Shale formation to clarify whether these compounds can reach shallow groundwater aquifers and affect local water quality after injection into deep shale horizons (Drollette et al. 2015). The authors detected hydrocarbons from diesel in 23 of 41 analyzed groundwater samples at concentrations ranging up to 157.6 µg/L. BTEX concentrations were below the U.S. EPA MCLs for drinking water. The presence of bis (2-ethylhexyl) phthalate was demonstrated, a disclosed HF additive, which was not detectable in geogenic water samples and field blanks (Drollette et al. 2015). Inorganic chemical fingerprinting of deep saline groundwater, analysis of characteristic noble gas isotopes, and studies of spatial relationships of shale gas allowed the differentiation between naturally occurring saline groundwater and contaminated water, e.g., by accidental leaks (Drollette et al. 2015). The authors concluded that contamination of groundwater was more likely to be due to the accidental release of chemicals derived from the surface than to subsurface flow of the injected organic compounds.

## Contamination by leakage of production water pipelines

In Lower Saxony, the center of natural gas production in Germany, leakage of production water pipelines has been repeatedly reported, with the consequence of contaminations of soil and groundwater (LBEG 2019a). After several incidents of leakage of the polyethylene pipelines, in April 2011, the Saxony Authority for Mining, Energy and Geology ordered that the responsible company should prove that the used synthetic pipeline was tight under the expected mechanical, thermic and chemical stresses. As a consequence of this check completed in May 2012, approximately 44 of a total of 740 km pipeline had to be decommissioned. However, even after May 2012, various cases were reported on the discharge of deposit water or wet oil, leading to contamination of soil and groundwater. Contamination was usually restricted to the direct environment of leakage and normally only a few square meters of soil were affected. The volumes of produced water were generally less than 2 m^3^. Adverse effects of residents in the neighborhood of leaking pipelines were not reported (LBEG 2019a).

## Contamination of surface waters and consequences for drinking water quality

Ground- and surface water represent important resources for the generation of drinking water. Therefore, it may lead to critical situations if surface water is used for both disposal of flow-back/production water and generation of drinking water. Direct injection or indirect discharge of inadequately cleared production water from oil or gas production into surface water represents a potential risk. Direct injection of production water is still of high relevance in the USA. Although this route of water disposal is generally prohibited according to the “oil and gas extraction effluent guidelines and standards” of the U.S. EPA (40 U.S. Code of Federal Regulations, CFR, 125.3, subpart C), exceptions are allowed in the arid zones of the USA west of the 98th longitude (U.S. EPA 2016a). In the latter case, surface water discharge of produced water has a portion of all disposal practices between two (California) and ten percent (Colorado). For Texas and Utah, five and six percent, respectively, are reported whereas in Arizona, North Dakota and Oklahoma, the portion amounts to zero percent.

Compounds in production water/flow-back depend on the specific geological formations (Shrestha et al. 2017; Luek and Gonsior 2017). Production water often contains inorganic compounds, such as bromide, chloride, iodide, barium, calcium, copper, iron, magnesium, strontium, sulfur, arsenic, selene and ‘Naturally Occurring Radioactive Material’ (NORM) (Ferrar et al. 2013; Warner et al. 2013; Weaver et al. 2016). Indicators for the analysis of a possible contamination of surface water by HF wastewater are chloride and ‘Total Suspended Solids’ (TSS) (Gagnon et al. 2016).

Flow-back/production water also contains numerous organic compounds, particularly benzene and BTEX. Importantly, sewage plant outflow, municipal facilities, as well as specialized plants for industrial wastewater, still contain toxic metals, e.g., barium and strontium, radioactive elements, e.g., radium isotopes, benzene, toluene, and high salt concentrations (TDS up to 254,000 mg/L) despite treatment (Ferrar et al. 2013). Bromide in HF wastewater and its environmental relevance have been intensively studied because it is only partially removed by sewage plants (Warner et al. 2013). Therefore, the disposal of cleared water from sewage plants into surface water may lead to increased bromide concentrations. Bromide concentrations in production water of the Marcellus formation of 1283, 787 and 744 mg/L have been reported, while 643 ± 201 mg/L (standard deviation) was measured in the effluent wastewater treatment plants. Other studies also reported relatively high bromide concentrations in the flow-back, ranging between 16 and 1190 mg/L and < LOD and 613 mg/L (Hayes 2009; Haluszczak et al. 2013). In unpolluted freshwater, bromide concentrations are much lower, ranging between < LOD and 0.5 mg/L. In seawater, concentrations are reported to range between 65 and 80 mg/L (WHO 2009).

Human toxicity of bromide after chronic oral uptake is considered to be low. A drinking water value of 6 mg bromide/L drinking water has been derived by the WHO, based on an Acceptable Daily Intake (ADI) of 0.4 mg/kg body weight/day for a person of 60 kg, consuming 2 L drinking water per day and the assumption that 50% of bromide exposure occurs via drinking water (WHO 2009). However, bromide can be transformed into more toxic compounds during drinking water treatment. Increased bromide concentrations in water can lead to increased levels of Brominated Disinfection By-products (DBP) and thus far unregulated compounds, such as halonitromethane, haloamide and haloacetonitriles (Parker et al. 2014; Weaver et al. 2016). For example, drinking water disinfection with elementary chlorine, chloramine and ozone in the presence of organic compounds in water may lead to the formation of trihalomethane and halogenated acetic acids. For some of these compounds, health-based guidance values have been derived, e.g., 100 µg/L for bromoform and dibromochloromethane and 60 µg/L for bromodichlormethane in drinking water. Drinking water disinfection with ozone may lead to oxidation of bromide to the mutagenic and carcinogenic bromate, for which 10 µg/L has been set as a provisional guideline value (WHO 2017). This guideline value is provisional because of limitations in available analytical and treatment methods. A health-based value of 2 μg bromate/L can be derived using an upper-bound estimate of cancer potency for bromate of 0.19 per mg/kg body weight per day, based on low-dose linear extrapolation. A one-stage Weibull time-to-tumor model was applied to the incidence of mesotheliomas, renal tubule tumors and thyroid follicular tumors in male rats given potassium bromate in drinking water. The concentration of 2 µg/L is associated with the upper-bound excess cancer risk of 10^−5^ (WHO 2009). However, WHO (2017) states also “emerging evidence” pointing “to rapid decomposition of bromate in the gastrointestinal tract, blood and liver, which supports a non-linear dose–response relationship at low doses”.

Quantitative aspects of the formation of brominated and iodinated trihalomethane (THMs) and haloacetonitriles (HANs) in mixtures of HF wastewater and surface water have been analyzed for the Ohio and Allegheny rivers and the Marcellus formation (Parker et al. 2014). Even chlorination of a mixture with as little as 0.01% HF wastewater leads to the formation of THMs and HANs, which was more pronounced in both substance classes at a level of 0.03% wastewater. Chloramine reduces HAN formation and regulates THM formation. In river water affected by municipal wastewater treatment processes, a HF wastewater percentage of 0.1% increases the formation of *N*-nitrosodimethylamine at iodide levels of 54 ppm during the reaction with chloramine. A significant increase in bromate formation was observed at a fraction of 0.01–0.03% HF wastewater. The authors recommend an alternative modified disinfection strategy that includes the change of chlorine to chloramine, the general prevention of the introduction of HF wastewater in surface waters, or the removal of the salt load in the wastewater.

The direct discharge of HF wastewater has already been prohibited in Pennsylvania (in 2011) since this state was particularly affected by contamination of surface waters due to HF. Nevertheless, increased concentrations of bromide were also observed after this ban. Also, the isotope ratios of ^87^Sr/^86^Sr and ^228^Ra/^226^Ra in receiving waters of Pennsylvania suggested that the HF outflow was further directly discharged into surface waters or that clearance of wastewater was insufficient (States et al. 2013).

Concentrations of toxic and radioactive elements in produced water have been reported to correlate with salinity, which may be explained by the geochemical properties of rock layers and deep water (Vengosh et al. 2014). Accumulation of radioactive elements has been observed in sediments of rivers and lakes, where produced water has been discharged. ^226^Ra levels in sediments of rivers of 544–8759 Bq/kg were detected that were approximately 200-fold higher compared to sediments upstream of the position of the discharge of production water (Warner et al. 2013). The general background radioactivity ranged between 22 and 44 Bq/kg.

Meanwhile, U.S. EPA modified its “Oil and Gas Extraction Effluent Guidelines and Standards (40th U.S. Code of Federal Regulations (CFR) Part 435)”. Under Subpart C, they prohibit the discharge of unconventional natural gas effluents to municipal sewage treatment plants (US EPA 2016b).

## Adverse soil alterations

### Local contamination of soil at production sites

Local contamination can result from the disposal of excavated material from drilling of the natural gas borehole close to the gas well site in oil sludge pits and from improperly performed maintenance and cleaning works on the site. Possible consequences are contaminations of groundwater, point pollution of soil and toxic air emissions. Another pathway of point pollution is the accidental HF wastewater surface spill on soil.

Oetjen et al. (2018) studied the ability of surfactants in HF wastewater to be transported through agricultural soil and to mobilize metals in soil using column experiments. Of the 27 surfactants (including polyethylene glycols, benzalkonium chlorides, and alkyl ethoxylates) known to be present in the wastewater samples or of their transformation products, none were measured in leachate samples. Conversely, copper, lead, and iron were mobilized at environmentally relevant concentrations: dissolved copper and lead concentrations increased from 40 μg Cu/L and from below the detection limit of 2 μg Pb/L during simulated rain events up to 300 μg Cu/L and 12 µg Pb/L in leachate.

## Local soil contamination in Germany: mercury, PAHs and benzene

**Fig. 4:**
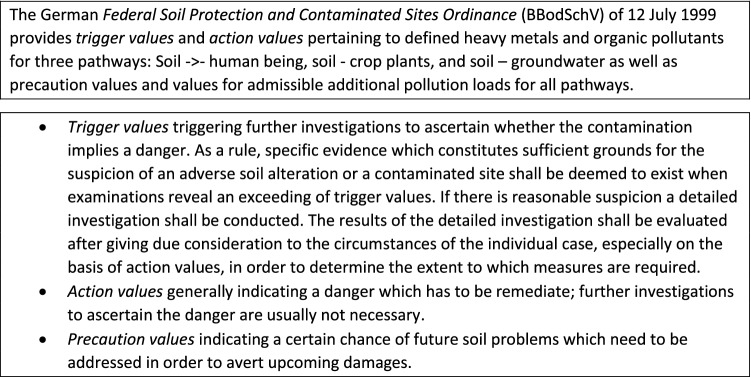
Categories of assessment values corresponding to the German Federal Soil Protection and Contaminated Sites Ordinance

Natural gas in Northern Germany may contain mercury in concentrations of up to 4.5 mg/m^3^. Contamination of soil with mercury would be a major concern since it is neuro-, nephro-, and reprotoxic. In addition to mercury, PAHs and benzene should also be considered as toxicologically relevant contaminants. Therefore, soil and sediment samples in the vicinity of 211 active natural gas production sites in Lower Saxony (Northern Germany) were systematically analyzed between July 2015 and May 2017 (Schneider et al. 2018). The analytical program comprised metals, heavy metals including mercury, organic compounds (PAH, C_10_–C_4_ hydrocarbons, BTEX, TOC, PCDD/PCDF), and specific radioactivity (radioactivity per unit mass of soil). In total, 2146 soil and 145 sediment samples were analyzed, leading to a large and representative dataset for Lower Saxony. The toxicological assessment of the measured soil concentrations was performed according to the systematics of the Federal Soil Protection and Contaminated Sites Ordinance (Fig. 4). The used assessment values are given in Table 8. For three of the analyzed 211 well sites, the action value for the pathway *soil*–*agricultural plants* for mercury of 2 mg Hg/kg soil were exceeded, with concentrations of 2.01, 2.94 and 8.14 mg/kg in three separate samples. Here, remediation was required. The trigger value for benzo(a)pyrene of 1 mg/kg soil was exceeded for eleven sites for the pathway soil–human being. At eleven sites, assessment values for mercury were exceeded in sediments from ditches. Precautionary values for mercury that depend on the type of soil range between 0.1 and 1.0 mg/kg were exceeded in 838 samples. Due to its carcinogenic properties and recent epidemiological evidence of locally increased hematological cancers, the analysis of benzene was of particular relevance. At two sites, benzene concentrations were 0.07 mg/kg, which only slightly exceeded the limit of quantification of 0.05 mg/kg. When precautionary values are exceeded, the operators of the plants must take adequate measures to avoid or minimize contaminations in future. Although several precautionary values and even action values were locally exceeded, no evidence of an extensive area-wide contamination was obtained in the environment of the studied production sites in Lower Saxony.

**Table 8:** Soil and groundwater screening values for specific oil and natural gas pollutants

	Mercury	Benzo(a)pyrene	Benzene	Alkylated benzenes (BTEX)	Benzene	Hydrocarbons	PAHs^a^	Naphthalene and methylnaphthalenes
	Soil (mg/kg dry matter)^b^			Groundwater (µg/L)^c^				
Precautionary values								
Soil type clay	1 (0.3)							
Soil type loam/silt	0.5 (0.3)							
Soil type sand	0.1 (0.2)							
Precautionary value at TOC ≤ 4%		(0.3)						
Precautionary value at TOC > 4–9%		(0.6)						
Precautionary value at humus content > 8%		1						
Precautionary value at humus content ≤ 8%		0.3						
Trigger values								
Playgrounds	10	2 (0,5)						
Residential areas	20	4 (1)	0.1					
Parks and recreational facilities	50	10 (1)						
Land used for industrial and commercial purposes	80	12 (5)	0.4					
Insignificance thresholds for groundwater				20	1	100	0.2	2

^a^16 EPA PAHs without naphthalene and methylnaphthalenes

^b^Federal Soil Protection and Contaminated Sites Ordinance (BBodSchV) (Federal Government 1999), values in brackets: draft “Mantelverordnung” (BMU 2017)

^c^LAWA (2004/2017) Determination of insignificance thresholds for groundwater

Recently, an epidemiological study reported an increase in early childhood leukemia in the environment of a former production site (EKN 2016). Therefore, a toxicological evaluation of environmental contaminations in this region was performed (Wollin 2016b, 2017a, b). The plant site was used as a transshipment point for crude oil and for crude oil processing between 1950 and 1995. Currently, the direct proximity of the former plant is dominated by residential buildings. The focal point of the partly considerable soil pollution with benzene, BTEX and hydrocarbons was along the hazard pathway soil–groundwater. Guideline values (Table 8) for compounds originating from crude oil in deeper soil and in groundwater were sometimes massively exceeded. Locally very high concentrations were detected for benzene (up to 1840 µg/L), ∑ BTEX (up to 1200 µg/L), hydrocarbons (up to 25,000 µg/L), polycyclic aromatic hydrocarbons (up to 28.5 µg/L) and naphthalene (up to 100 µg/L). Nevertheless, these locally high values did not lead to increased risks for residents because the groundwater of the core area was not utilized and samples of domestic water from neighboring residential estates were not contaminated. By contrast, the parameters measured in the topsoil for the direct pathway soil–human fell below the guide values of BBodSchV in all subareas or were below the limit of quantification. Importantly, pooled soil samples did not exceed trigger values for children’s playgrounds (Wollin 2016b, 2017a, b).

## Large-scale contamination of soil

The use of produced water for agricultural purposes is permitted in the West of the USA, e.g., in California (Stringfellow et al. 2017b). Production water is applied for irrigation of agricultural areas, watering places for livestock, and for groundwater recharge in natural wetlands. nds in flow-back/production water can, in principle, cause an increased risk of adverse effects in humans. HF-associated compounds could be taken up by agricultural plants or contaminate the plants via their surface. Moreover, watering of agricultural areas by production water may lead to contamination of local groundwater. In the case of non-biodegradable substances, there is a concern of (bio)accumulation. Finally, occupational exposure of farmers to production water should be considered as a further possible risk. Considering the increasing interest in using production water for agricultural purposes, these potential risks should be systematically evaluated (Stringfellow et al. 2017b). Another cause of agricultural soil contamination can be spills of HF fluids or frac chemicals, and flow-back/produced water. When investigating spills with only a limited range of inorganic parameters and substances such as BTEX, and total petroleum hydrocarbons (TPHs), there is a danger that other substances will not be identified and the extent of the damage not fully recognized (McLaughlin et al. 2016). So far, little is known about the environmental behavior of frac chemicals and compounds in production water, particularly their sorption behavior, transformations and interactions. However, this knowledge is essential for the assessment of possible human risks due to the exposure paths from soil to agricultural plants, as well as from soil to groundwater. McLaughlin et al. (2016) examined the environmental fate of the widespread used HF chemicals glutaraldehyde, polyethylene glycol surfactants, and polyacrylamide-based commercial friction reducers. The polyethylene glycol tensides were found to be completely biodegradable on agricultural soil within 42–71 days. Their biodegradation was reduced in the presence of the biocidal product, glutaraldehyde. Salts, at concentrations typically occurring in production water, strongly reduced their biodegradation. The availability of glutaraldehyde in soil is reduced by adsorption to soil components; the biocide itself was completely biodegraded within 33 and 57 days. Polyacrylamide, which is used in frac fluids for friction reduction, interacts with glutaraldehyde and reduces its biodegradation (McLaughlin et al. 2016). Surfactants may increase the mobility of other organic HF additives through co-solvent effects and possibly solubilize otherwise immobile metals in the soil.

## Contamination of air

### Air pollution

#### Data from the USA

In the USA, a high fraction of greenhouse gas and volatile organic compounds (VOC) are due to oil and gas production (including conventional production). It should be noted that ~ 1.1 million oil and gas wells were in use in 2009 (U.S. EPA 2012). Frac operations are performed in ~ 11,400 new wells per year. Emissions from U.S. oil and gas activities in 2005 have been reported to be 321, 318, 510 and 619 kT for NO_*x*_, CO, NMVOCs (non-methane volatile organic compounds), and SO_2_, respectively (U.S. EPA 2019). By 2017, these numbers increased to 650, 637, and 2853 kT, respectively, whereas SO_2_ decreased to 87 kT. In particular, the large increase in NMVOCs, including hazardous air pollutants, is of potential concern. Natural gas systems were the second largest anthropogenic source category of methane emissions in the U.S. in 2017, after agriculture. Overall, natural gas systems emitted 165.6 MMT CO_2_ Eq. of methane in 2017, a 14% decrease compared to 1990 emissions (U.S. EPA 2019). Between 2013 and 2017, the methane emissions from natural gas systems reported for the sum of the processes *field production*, *processing*, *transport* and *storage*, as well as distribution, were virtually unchanged (each with 165.6 MMT CO_2_ Eq. in 2013 and 2017; as well as 165.1, 167.2, and 165.7 MMT CO_2_ Eq., respectively, in 2014, 2015 and 2016). Moreover, abandoned gas and oil drilling sites may contribute to methane emission. The fraction of abandoned sites of the total anthropogenic emission of methane has been estimated to range between 4 and 7%.

Differentiation of emissions from oil and gas production and other sources, such as agriculture, traffic or landfill sites, is possible by specific patterns of VOCs (Gilman et al. 2013). It is possible to clearly identify VOC emissions that are due to oil and gas production based on the lead compounds, propane and ethyne. Using this approach, it can be estimated that approximately 55% of total VOC-OH reactivity in the USA is due to oil and gas production.

### Air contamination by hydraulic fracturing

Similar to conventional oil and gas production, HF processes also lead to contamination of ambient air with methane, further aliphatic hydrocarbons, such as C_2_–C_5_, alkanes, VOCs, such as BTEX, hydrogen sulfide, n-hexane, and formaldehyde (Macey et al. 2014; Vinciguerra et al. 2015; Allen 2016). Recently, it has been reported that 143 air contaminants may be released due to HF (Elliott et al. 2017a). Hazard assessment by the IARC concerning carcinogenicity is available for only 20% of these compounds. Of 29 potential air contaminants, 20 compounds were known human carcinogens (IARC group 1), probably carcinogenic for humans (group 2A), or possibly carcinogenic for humans (group 2B).

Further air contaminants are generated by the peripheral plant components, including particulate matter, NO_*x*_, precursors of ozone and polycyclic aromatic hydrocarbons (Paulik et al. 2016). The following activities are known to contribute to air contamination at oil or gas drilling sites:

- Preparation of the drilling site including road connections.
- Drilling of the well.
- Truck traffic for delivery and disposal of materials.
- Removal of acid gases and water from gas; separation of natural gas from other hydrocarbons.
- Operation of compressor stations to enable the transport of natural gas into transport pipelines.
- Preprocessing of crude oil prior to refinery.
- Flaring of gas.
- Volatile emissions from leaks.
- Gas release by pressure compensation.

The most important sources of air contamination are summarized in Table 9. Examples of toxic compounds reported to be released into ambient air during HF in the USA are listed in Table 10. NO_*x*_ and SO_*x*_ emissions have been reported to be higher during the development of the drilling site compared to the production phase (Colborn et al. 2014; Litovitz et al. 2013). Similar observations have been made for particulate matter (PM2.5 and PM10). Analysis of shale gas production sites in North Texas showed an increase in ozone concentrations by 8% at gas production sites compared to control sites (Ahmadi and John 2015).

**Table 9:** Sources of emissions of air contaminants by HF (modified from Robinson 2014)

Source	Air pollutant				Data quality
	NO_X_	VOC	PM	Other toxic substances	
Well development					
Drilling rigs	•	◦	•	•	Medium
Frac pumps	•	◦	•	•	Medium
Truck traffic	•	◦	•	•	Medium
Completion venting		•		•	Poor
Frac ponds		◦			Poor
Gas production					
Compressor stations	•	•	◦	•	Medium
Wellhead compressors	◦	◦	◦	◦	Medium
Heaters, dehydrators		◦	◦	◦	Medium
Blowdown venting		◦		◦	Poor
Condensate tanks		•		◦	Poor
Fugitives				◦	Poor
Pneumatics		◦		◦	Poor

• Major source, ◦ minor source

**Table 10:** Ambient air contamination in areas of the USA with HF activities

References	Region	Compound/compound group	Concentration (µg/m^3^, unless differently specified)	Number of samples	Duration of sampling	Year of sampling
Bunch et al. (2014)	Barnett shale region (Texas)	Benzene	0.0053–28	101318	1-h mean 1-year mean	2011
			0.25–0.77			
		Ethylbenzene	0.0054–15	101044		
			0.10–0.35			
		*m*/*p*-Xylene	0.0054–59	101141		
			0.21–0.41			
		*o*-Xylene	0.0054–21	101196		
			0.09–0.35			
		*n*-Hexane	0.0117–263	100044		
			0.79–2.67			
		Toluene	0.0054–653	101216		
			0.48–2.29			
		Benzene	0.064–11	1164	24-h mean 1-year mean	
			0.52–1.15			
		Ethylbenzene	0.043–1.9	1164		
			0.15–0.45			
		*m*/*p*-Xylene	0.043–8.4	1164		
			0.42–1.02			
		*o*-Xylene	0.043–2.1	1164		
			0.13–0.39			
		*n*-Hexane	0.035–302	1164		
			0.38–9.11			
		Toluene	0.113–191	1164		
			1.03–5.01			
Colborn et al. (2014)	Garfield County (Colorado)	Ethane	3.6–118.0 ppb_V_	48	4–6 h	July 2010–October 2011
		Propane	1.1–46.7 ppb_V_			
		Toluene	0.4–4.3 ppb_V_			
		Isopentane	0.4–7.3 ppb_V_			
		1,2,4-Trimethylbenzene	0.2–0.3 ppb_V_			
		Benzene	0.3–1.1 ppb_V_			
		Ethanol	3.2–19.4 ppb_V_			
		Methanol	12.1–30.6 ppb_V_			
		Formaldehyde	0.3–2.4 ppb_V_	43		
		Hexanal	0.1–0.2 ppb_V_			
		Isoprene	0.4–0.7 ppb_V_	48		
		Methylene chloride	2.7–1730.0 ppb_V_			
		*m*-/*p*-Xylene	0.2–0.7 ppb_V_			
		Tetrahydrofuran	2.1 ppb_V_			
		*n*-Hexane	0.3–3.0 ppb_V_			
		*n*-Heptane	0.3–1.4 ppb_V_			
		*n*-Octane	0.2–0.8 ppb_V_			
		*n*-Nonane	0.2–0.3 ppb_V_			
		2-Butanone (mek)	2.3–5.1 ppb_V_			
		Acetone	3.4–28.3 ppb_V_			
		Crotonaldehyde	0.1–3.0 ppb_V_			
Ethridge et al. (2015)	Barnett Shale/Texas, Dallas Hinton	Benzene	0.19 ppb_V_	91539	1 h	2012
	Barnett Shale/Texas, Fort Worth Northwest		0.19 ppb_V_	64372		
	Barnett Shale/Texas, Dish Airfield		0.15 ppb_V_	20.723		
	Barnett Shale/Texas, Eagle Mountain Lake		0.11 ppb_V_	20638		
	Barnett Shale/Texas, Decatur Thompson		0.16 ppb_V_	17205		
	Barnett Shale/Texas, Flower Mound Shiloh		0.13 ppb_V_	15976		
	Barnett Shale/Texas, Everman Johnson Park		0.13 ppb_V_	10958		
	Barnett Shale/Texas, Kennendale Treepoint Drive		0.14 ppb_V_	3926		
	Barnett Shale/Texas, Midlothian Old Fort Worth		0.25 ppb_V_	824	24 h	2009–2012
	Barnett Shale/Texas, Denton Airport South		0.23 ppb_V_	736		
	Barnett Shale/Texas, Dallas Hinton		0.27 ppb_V_	717		
	Barnett Shale/Texas, Greenville		0.24 ppb_V_	579		
	Barnett Shale/Texas, Fort Worth Northwest		0.26 ppb_V_	547		
	Barnett Shale/Texas, Grapevine Fairway		0.19 ppb_V_	536		
	Barnett Shale/Texas, Kaufman		0.20 ppb_V_	519		2009–2012
	Barnett Shale/Texas, Italy		0.17 ppb_V_	313		
	Barnett Shale/Texas, Johnson County Luisa		0.18 ppb_V_	122		
Macey et al. (2014)	Fremont (Wyoming) 5 m distance to separators	H_2_S	590	1	2–3 min	k. A.
		Benzene	2.200	1		
		Toluene	1.400	1		
		Ethylbenzene	1.200	1		
		Xylenes	4.100	1		
		*n*-Hexane	22.000	1		
	Park (Wyoming) 25 m distance to separators	H_2_S	91	1		
		Benzene	110.000	1		
		Toluene	270.000	1		
		Xylenes	135.000	1		
		*n*-Hexane	1,200,000	1		
	Cleburne, Faulkner, and Van Buren (Arkansas) 30–355 m distance to separartors	Formaldehyde	8.5–48	13	8 h	
		1,3-Butadiene	8.5	8	2–3 min	
	Susquehanna (Pennsylvania) 230–790 m distance to compressor	Formaldehyde	7.6–61	10	8 h	
	270 m Entfernung zu PIG Launch	Benzene	5.7	4	2–3 min	
McKenzie et al. (2012)	Garfield (Colorado) Region with HF development	1,2,3-Trimethylbenzene	0.022–0.85	163	24 h	2008–2010
		1,2,4-Trimethylbenzene	0.063–3.1			
		1,3,5-Trimethylbenzene	0.024–1.2			
		1,3-Butadiene	0.025–0.15			
		Benzene	0.096–14			
		Cyclohexane	0.11–105			
		Ethylbenzene	0.056–8.1			
		Isopropylbenzene	0.020–0.33			
		Methylcyclohexane	0.15–24			
		*m*-Xylene/*p*-xylene	0.16–9.9			
		*n*-Hexane	0.13–25			
		*n*-Nonane	0.064–3.1			
		*n*-Pentane	0.23–62			
		*n*-Propylbenzene	0.032–0.71			
		*o*-Xylene	0.064–3.6			
		Propylen	0.11–2.5			
		Styrene	0.017–3.4			
		Toluene	0.11–79			
	Garfield (Colorado) Gas well, 40–152 m distance to the drilling site	1,2,3-Trimethylbenzene	0.055–12	24	24 h	
		1,2,4-Trimethylbenzene	0.44–83			
		1,3,5-Trimethylbenzene	0.33–78			
		1,3-Butadiene	0.068–0.17	16		
		Benzene	0.94–69	24		
		Cyclohexane	2.21–200			
		Ethylbenzene	0.25–230			
		Isopropylbenzene	0.0–4.8			
		Methylcyclohexane	3.1–720			
		*m*-Xylene/*p*-xylene	2.0–880			
		*n*-Hexane	1.7–255			
		*n*-Nonane	1.2–300			
		*n*-Pentane	3.9–550			
		*n*-Propylbenzene	0.098–12			
		*o*-Xylene	0.38–190			
		Propylene	0.16–1.9			
		Styrene	0.23–5.9			
		Toluene	2.7–320			
Paulik et al. (2016)	Carroll County (Ohio)	Σ PAH (62 single compounds, means)	7.4 ng/m^3^ (< 161 m distance from an active well)	5	3–4-weeks	2014
			8.4 ng/m^3^ (< 161 m–1.61 km distance from an active well)	12		
			6.7 ng/m^3^ (> 1.61 km distance from an active well)	6		
		Benzo(a)pyrene; Benzo(a)pyrene_eq_ (means)	1.4 × 10^−5^ ng/m^3^; 13 × 10^−4^ ng/m^3^ (< 161 m distance from an active well)	5		
			7.1 × 10^−6^ ng/m^3^; 11 × 10^−4^ ng/m^3^ (< 161 m–1.61 km distance from an active well)	12		
			2.4 × 10^−6^ ng/m^3^; 8.6 × 10^−4^ ng/m^3^ (> 1.61 km distance from an active well)	6		
Rich and Orimoloye (2016)	Dallas (Fort Worth Metroplex), residential areas	Benzene	0.6–592 ppb_V_ 2.93–2.900.2 ppb_V_	50	24 h 1 h	2008–2010
		Benzene max.	592 ppb_V_	50	24 h	
		Toluene max.	276 ppb_V_			
		Ethylbenzene max.	113 ppb_V_			
		*m*-Xylene/*p*-xylene max.	221 ppb_V_			
		*o*-Xylene max.	39.4 ppb_V_			
		1,3,5-Trimethylbenzene max.	9.95 ppb_V_			
		1,2,4-Trimethylbenzene max.	60.4 ppb_V_			
		Styrene max.	43.4 ppb_V_			
		Propylbenzene max.	23.5 ppb_V_			

A critical aspect concerning emissions of HF processes is that most emitted organic toxic compounds are not regulated. This is the case for the National Ambient Air Quality Standards (NAAQS) of the U.S. EPA according to the Clean Air Act. Here, the six so-called ‘Criteria Air Pollutants’ are carbon monoxide, ozone near the surface, nitrogen dioxide, particulate matter, sulfur dioxide, and lead. Since the criteria for organic air pollutants are not available in the NAAQS, the Reference Concentrations (RfCs) of the U.S. EPA (Integrated Risk Information System, IRIS) are frequently used. For carcinogenic compounds, the inhalation MRLs of the Agency for Toxic Substances and Disease Registry (ATSDR) may be applied (Macey et al. 2014). However, these scientifically derived values are not legally binding in the U.S. and, therefore, their current national legal regulations of toxic organic air emissions are not sufficiently comprehensive for carcinogenic compounds.

In the European Union, benzene and polycyclic aromatic compounds, with benzo(a)pyrene as a lead compound, have been regulated in the ambient air with threshold values of 5 µg/m^3^ and 1 µg/m^3^, respectively (Lilienblum and Wollin 2019). Health risks for residents in the neighborhood of HF sites still are discussed controversially. Some studies reported an increased cumulative cancer risk associated with benzene, trimethylbenzene, xylene and aliphatic hydrocarbons (McKenzie et al. 2012; Macey et al. 2014; Rich and Orimoloye 2016); however, others did not confirm these associations (Bunch et al. 2014; Ethridge et al. 2015; Paulik et al. 2016). The discrepancies may be due to high spatiotemporal variability of air concentrations during sampling (Macey et al. 2014). The challenge of adequate sampling for analysis of air contamination has already been discussed (Brown et al. 2014; Haley et al. 2016).

To adequately consider the above-described variability a specific spatially temporally resolved analysis of exposure is required, which has been applied by Ethridge et al. (2015) in the Barnett Shale in Texas. The authors used infrared cameras to obtain an overview over regions of high hydrocarbon concentrations in the air. Based on these data, locations for air sampling were identified to determine concentrations of 85 individual compounds by gas chromatography. For the final analysis more than 4.7 million individual data points were available for short- (means of 1 h, 24 h, 7 days) and long-term (1 year and longer) analyses. In three of 1299 short-term samples, concentrations of benzene, n-heptane and n-octane exceeded short-term assessment values. Moreover, the odor thresholds of cyclohexane, isopentane, *m*- and *p*-xylene, methylcyclohexane, *n*-hexane, *n*-heptane and n-pentane were exceeded. However, the mean values of long-term exposure levels did not exceed the respective threshold values (Ethridge et al. 2015). The authors concluded that benzene and n-hexane might be considered as particularly relevant for the evaluation of health risks. A similar result was obtained by an analysis of emission levels in the Barnett Shale, where a similarly comprehensive study was performed (Bunch et al. 2014). None of the analyzed VOCs exceeded short-term assessment values; and only in case of 1,2-dibromomethane the assessment value for chronic exposure was exceeded. Of note, the authors discussed that 1,2-dibromoethane emission was not caused by the HF procedure. According to TCEQ (2011), 1,2-dibromoethane is not a VOC that is reasonably expected to be associated with shale gas operations, but it was used as a lead scavenger in aviation fuel and off-road applications in automobile racing (U.S. EPA 2009). Significant concentrations of lead scavengers continue to persist at many old leaded gasoline spill sites.

In a further study, air samples in the neighborhood of HF development and production sites were taken by trained residents at locations identified through systematic observation of the HF operations and air impacts over the course of resident daily routines (Macey et al. 2014). Residents responded by sampling to operational conditions, odor events, and the onset of acute health symptoms. Grab air samples (*n* = 35) were taken into 10-L Tedlar bags and were analyzed for 75 VOCs, including BTEX, acrylonitrile, methylene chloride, hexane, and heptane. Additionally, formaldehyde and 20 sulfur compounds (including hydrogen sulfide and carbonyl sulfide) were analyzed using passive sampling. In 16 of the 35 Tedlar bag samples and 14 of the 41 passive samples, eight VOCs exceeded the assessment values of the ATSDR and/or the U.S. EPA IRIS RfCs. Benzene, formaldehyde and hydrogen sulfide were the most frequent compounds that exceeded short-term and chronic assessment values.

A study in Garfield County/Colorado, analyzed health risks as a consequence of air emissions due to HF in unconventional gas resources (McKenzie et al. 2012). Air samples were taken in rural residential estates or farms (*n* = 163) located either ≤ 0.8 km or > 0.8 km from gas wells, and 78 hydrocarbon compounds were analyzed. The data were used to calculate hazard indices (HIs). HIs were higher for residents living less than 0.8 km from wells compared to individuals living further away. The highest non-cancer HI of five was obtained for sub-chronic exposure during well completion for residents living ≤ 0.8 km from the gas well. This high HI was attributed to exposure to trimethylbenzene, xylenes and aliphatic hydrocarbons. Evaluation of the chronic risk accounts for exposure to air emissions from well completions and emissions from the production phase. For chronic exposure, non-cancer HIs of 1.0 were obtained for residents ≤ 0.8 km from wells and 0.4 for residents > 0.8 km. For carcinogens, the lifetime cancer risk for each compound was derived by multiplying estimated exposure concentration by the inhalation unit risk. The cumulative cancer risk was estimated by the addition of the cancer risks for individual compounds. Risks are expressed as excess cancers per 1 million population based on exposure over 30 years. Cumulative cancer risk was estimated to be 10 × 10^−6^ and 6 × 10^−6^ for residents living ≤ 0.8 km as well as > 0.8 km from the wells.

A quantitative assessment of cancer risk due to 62 analyzed PAHs from HF emissions was performed in a rural community (Paulik et al. 2016). The obtained PAH patterns indicated that the analyzed PAHs were predominantly of petrogenic origin. The highest concentrations of benzo(a)pyrene, phenanthrene and the highest carcinogenic potential of PAH mixtures, determined as the sum of benzo(a)pyrene-equivalents of the detected PAHs, were obtained in the direct neighborhood of active wells. At these sites of maximal exposure, the additional carcinogenic lifetime risk was estimated to be 0.04 × 10^−6^ and, therefore, fell below the additional carcinogenic lifetime risk of 1 × 10^−6^ that the U.S. EPA considered as acceptable.

McMullin et al. (2018) analyzed exposure to VOCs emitted from exploitation regions with HF in Colorado. The authors identified 56 VOCs and compiled 47 existing air monitoring datasets that measured these VOCs in 34 locations across the exploitation regions. Based on the measured air concentrations, acute and chronic exposures were estimated by comparing exposures to health guideline levels using maximum and mean air concentrations. Selecting acute and chronic non-cancer health guidance values followed a tiered approach using U.S. EPA’s RfCs and Unit Risk estimates in the first line. Acute and chronic non-cancer hazard quotients were below one for all individual VOCs at distances of 500 feet (0.152 km, the current setback distances from new wells in the state Colorado) or greater from production sites. Hazard indices combining exposures for all VOCs were slightly above one. The lifetime excess cancer risk estimates for benzene were between 1.0 × 10^−5^ and 3.6 × 10^−5^ and ethylbenzene was 7.3 × 10^−6^.

### Data from Germany

In Germany, 2.106 kt of methane were emitted in 2018, which corresponds to 52.642 kt CO_2_-eq. (Eionet 2020). These emissions changed only little since 2011. Similar to the USA, agriculture in Germany contributes the largest fraction (61.8%) of methane emissions. Emissions of methane due to oil, gas, iron and steel production in Germany have been estimated to be very low to negligible (UBA 2017; Eionet 2020).

Several pilot studies of exposure to VOCs and mercury due to HF in Germany have been performed. Data from a gas drilling site in Rotenburg (Wümme) obtained between July 2015 and March 2016, a period during which flaring of gas was also performed, did not exceed the EU-emission limit value of benzene (5 µg/m^3^) (LBEG 2016). The assessment values of TEX and the guidance value of mercury of 50 µg/m^3^ (LAI 2004) were also not exceeded (Table 11). Benzene air concentration ranges were clearly below the EU-emission limit value and values were in line with those that are typically observed in rural and urban regions of Germany (GAA Hildesheim 2019). Similar results were obtained near a gas production site in Bellen, Söhlingen in 2012, in which the arithmetic means of 6-monthly average values were 0.5 µg/m^3^, 0.6 µg/m^3^ and 0.5 µg/m^3^ at three sampling points. A relevant influence of plant operations on emissions was not observed in this study.

**Table 11:** BTEX and mercury analyses at the permanent measurement point MP 01 from July 2015 to March 2016 (modified according to LBEG 2016

Sample site MP 01 (20 °C; 1013,25 hPa)	Month	Year	Benzene (µg/m^3^)	Toluene (µg/m^3^)	Ethylbenzene (µg/m^3^)	Xylenes (µg/m^3^)	Mercury (ng/m^3^)
	July	2015	0.1	0.3	< 0.1	< 0.2	2.0 1.8
	August	2015	0.2	0.3	0.1	0.2	1.3 1.9
	September	2015	0.2	0.3	0.1	0.3	1.4 1.4
	October	2015	0.7	0.6	0.1	0.4	1.6 1.5
	November	2015	0.3	0.4	0.1	0.3	1.7 1.3
	December	2015	0.4	0.4	0.1	0.4	1.5 1.4
	January	2016	0.9	0.6	0.1	0.4	1.5 1.7
	Februar	2016	0.5	0.4	0.1	0.3	1.7 1.7
	March	2016	0.4	0.4	0.1	0.2	n.a.
Arithmetic mean			0.4	0.4	0.1	0.3	1.6
Assessment value			5^a^	5.000^b^	1.000^c^	100^d^	
				3.800^e^	260^f^	217^g^	
				30^h^		30^i^	50^k^

^a^Limit value (Directive 2008/50/EC); ^b^RfC (IRIS U.S. EPA 2020); ^c^RfC (IRIS U.S. EPA 2020); ^d^RfC (IRIS U.S. EPA 2020); ^e^chronic inhalation MRL (ATSDR 2019); ^f^chronic inhalation MRL (ATSDR 2019); ^g^chronic inhalation MRL (ATSDR 2019); ^h^Target value of the air purity guidelines (LAI 1997); ^i^Target value of the air purity guidelines (LAI 1997); ^k^Guidance value mercury (LAI 2004)

Epidemiological studies showed increased early childhood leukemias in the neighborhood of a cold-gas flare installation in Germany that was operated until 1989 (EKN 2016). Analyses in 1988 showed that benzene mass concentrations were exceeded by factors of 333–380 in the exhaust gas. Benzene mass flow was exceeded by factors of 4.0–4.5 (TÜV Hannover 1988). An immission analysis of BTEX was performed in 2016 (Wollin 2017a, b; ZUS LLG 2016), in which data were obtained for five assessment points that corresponded to directly adjacent residential estates (Table 12). The distance of these residential estates to the cold-gas flare was only 100–300 m. The predicted *total benzene load* in the immission estimated as the sum of the predicted additional benzene load in the immission and the background ranged between 1.42 and 4.82 µg/m^3^. The background level of 0.7 µg/m^3^ for rural areas (LAI 1992) was used to estimate the additional contribution of the cold-gas flare. This showed that the current, legally binding, maximum emission limit value of benzene (5 µg/m^3^) was not exceeded at any point of analysis. However, in the case of the maximally detected concentration of 4.82 µg/m^3^, the benzene threshold was almost reached. It should be considered that measurements of emissions in the ambient air could only be used for an approximate estimation of average exposure in relation to the distance from production sites. There may also be a variety of sources of emissions measured, not only the pollutants arising from gas or oil production (Zielinska et al. 2014), and they may not accurately describe the exposure of individuals.

**Table 12:** Additional BTEX load in the immission (ZUS LLG 2016)

BUP	Benzene (µg/m^3^)		Toluene (µg/m^3^)		Ethylbenzene (µg/m^3^)		Xylenes (µg/m^3^)	
	Min.	Max.	Min.	Max.	Min.	Max.	Min.	Max.
X_1_	3.62	4.12	0.44	0.61	0.02	0.05	0.02	0.06
X_2_	0.64	0.72	0.08	0.11	< 0.01	0.01	< 0.01	0.01
X_3_	0.82	0.94	0.10	0.14	< 0.01	0.01	0.01	0.01
X_4_	0.95	1.11	0.12	0.17	< 0.01	0.01	0.01	0.02
X_5_	1.75	2.19	0.22	0.33	0.01	0.02	0.01	0.03

## Hydraulic fracturing and human health risks

Quantitative assessment of toxicological risks of HF should include all upstream processes, such as production and transport of the required frac chemicals; onsite activities, such as storage of chemicals, production water; drilling and downstream processes, such as the processing of hydrocarbons and production water. Health risks for humans may also occur via environmental contamination or direct exposure at working places (Adgate et al. 2014; Goldstein et al. 2014).

In some cases, human health risks have been exclusively based on data of acute toxicity (Stringfellow et al. 2014, 2017a). However, human risk evaluation of HF-associated processes should not be limited to the analysis of the inherent acute and chronic toxicity of chemicals in frac fluids or components of the flow-back, i.e., the hazard of the individual compounds (Stringfellow et al. 2014, 2017a, b; Elliott et al. 2017a, b; Wattenberg et al. 2015; Webb et al. 2014; Bergmann et al. 2014; Meiners et al. 2012a, b; Meiners 2012; Yost et al. 2016). In fact, the identification of inherent toxicity (hazard) represents a first and essential step in risk evaluation (NRC 1983, 2009; Wattenberg et al. 2015). Here, substances with the properties carcinogenic, mutagenic [e.g., Kahrilas et al. 2015; Elliott et al. 2017a (review)] and toxic to reproduction/developmental toxicity (Webb et al. 2014; Elliott et al. 2017b) as well as compounds with endocrine activities or endocrine disruptors (Kassotis et al. 2014) are of central interest.

It should be kept in mind that risk characterization represents the final step of the risk assessment procedure and is the basis of risk management (Wollin and Illing 2014). This means that the sole presence of a hazardous compound at working places or in a specific environmental compartment does not necessarily mean a health risk for humans; rather, it should be linked to a specific exposure (Saunders et al. 2018). Risk characterization focusses on the question of whether a specific external exposure leads to internal concentrations at target cells of toxicity that are high enough to cause adverse effects.

An alternative to risk assessment is the precautionary principle (Commission of the European Communities 2000). The precautionary principle in risk management requires a scientific assessment as far as possible and the identification of the degree of scientific uncertainty. An assessment is required of the consequences that would occur if no action is taken. Once the results of the scientific risk assessment are available, persons concerned should be involved in the examination of precautionary measures.

The public discussion about HF focused on health risks caused by chemicals that can be part of frac fluids, additives or compounds in the flow-back, as well as produced water. Moreover, emissions have been increasingly considered. Assessment of human risks can be performed based on legal limit values or health guidance values. If such values are not available, further principles may be applied, such as thresholds of toxicological concern (TTC) (assessment values for compounds with unknown toxicity), or the precautionary concept of Health-related Indication Values (HRIV) in Germany (Dieter 2014).

### Epidemiological studies of adverse health effects associated with HF

Only few epidemiological studies have been performed to specifically analyze the health consequences of HF. The vast majority of studies focused on conventional oil and gas production. Recently, several review articles focusing on health and environmental risks of HF have been published. One review (Werner et al. 2015) identified more than 1000 studies published between 1995 and March 2014 (peer-reviewed and gray literature); after analysis and filtering using defined inclusion criteria, only 109 studies remained, of which only seven were considered as highly relevant. The inclusion criteria rank the strength of evidence of health impact that is related to/caused by environmental hazards released by HF activities, which included qualitative and/or quantitative studies. Most publications focus on the consequences of HF for water and air, while consequences for human health were deduced indirectly but were not directly proven. Moreover, most studies focus on acute and not chronic toxicity, such as cancer or reproductive toxicity. It was concluded that direct scientific evidence of associations between HF and adverse effects in humans is missing; vice versa, adverse effects can also not be excluded. This uncertainty leads to an unsatisfactory situation concerning public health (Werner et al. 2015).

A second review was performed based on 685 original publications with peer review, published between January 2009 and December 2015 (Hays and Shonkoff 2016). They identified reports of increased hazard and adverse effects in 84% of the studies on public health; positive associations or contamination was reported in 69% of the studies on water quality; increased emissions of air pollutants in 87% of studies about the quality of air (Hays and Shonkoff 2016). The authors critically discuss the limitations of their review, including the binary categorization of the analyzed studies and the lack of analysis of the quality of study design, methods and implementation. The assessment of the scientific literature provides a general understanding of the weight of the scientific evidence of possible health impacts and can be used to prioritize future research, and to provide an empirical foundation for policy decisions (Hays and Shonkoff 2016).

A third scoping review analyzed 216 studies with a scientific peer review published between 2000 and September 2017 that focused on health effects of HF in the USA (Wright and Muma 2018). The authors excluded studies that exclusively presented stakeholder perceptions and finally identified 18 publications that fulfilled their criteria. Unlike full systematic reviews or meta-analyses, the authors did not aim to evaluate the quality of the studies. Three of the 18 studies did not focus on toxicological and/or environmental toxicological aspects but addressed risks at working places due to the upstream production of proppants. Ten studies identified statistically significant associations between HF and specific human health issues; six reported evidence for associations, while two did not identify any association.

Five of six studies on maternal, neonatal and childhood health reported inconsistent results; whereas, a further one found no such relationship. In the retrospective cohort study of 15,451 live births in Southwest Pennsylvania from 2007 to 2010, no significant association of proximity and density of HF with prematurity was found (Stacy et al. 2015). A comparison between the most and the least exposed women, however, revealed lower birth weights (3323 ± 558 vs 3344 ± 544 g) and a higher incidence of the outcome small for gestational age (6.5 vs 4.8%, respectively; odds ratio 1.34; 95% CI 1.10–1.63). The clinical significance of the differences in birth weight among the exposure groups is unclear since a birth weight of less than 2500 g is usually considered to be critical. The findings further emphasize a more precise and accurate characterization of exposure over an extended time period to evaluate the potential health significance of HF (Stacy et al. 2015). A similar retrospective cohort study using electronic health record data on 9384 mothers living close to HF sites linked to 10,946 neonates from January 2009 to January 2013 found a positive relationship between HF activity and premature births that increased as mothers’ exposure to activity increased (fourth quartile odds ratio 1.4 (95% CI 1.0, 1.9) (Casey et al. 2016). Post hoc analysis identified a positive relationship between HF and physician-identified high-risk pregnancy. The inverse-distance squared model to characterize exposure incorporated distance to the mother’s home; dates and durations of well pad development, drilling, and hydraulic fracturing; and production volume during pregnancy (Casey et al. 2016). In a further retrospective cohort study of 124,842 births between 1996 and 2009 in rural Colorado, an increasing prevalence of congenital heart defects with an odds ratio of 1.3 for the highest tertile (95% CI 1.2, 1.5) has been estimated (McKenzie et al. 2014). Neural tube defects prevalence was associated with the highest tertile of exposure (OR 2.0; 95% CI 1.0, 3.9, based on 59 cases), compared with the absence of any gas wells within a 10-mile radius. Exposure was negatively associated with preterm birth and positively associated with fetal growth, although the magnitude of association was small. No association was found between exposure and oral clefts. An inverse distance weighted approach was applied to estimate maternal exposure which accounts for the number of wells within the 10-mile radius of the maternal residence, as well as distance of each well from the maternal residence. The authors concluded that greater specificity in exposure estimates is needed to further explore these associations (McKenzie et al. 2014). A retrospective birth cohort study among 158,894 women with a birth or fetal death from November 2010 to November 2012 in the Barnett Shale (North Texas) found increased adjusted odds of preterm birth associated with HF activity in the highest tertiles of the 0.5- (OR 1.14; 95% CI 1.03, 1.25), 2- (OR 1.14; CI 1.07, 1.22), and 10-mile (OR 1.15; CI 1.08, 1.22) metrics (Whitworth et al. 2017). Increased adjusted odds of fetal death were found in the second tertile of the 2-mile metric (OR 1.56; CI 1.16, 2.11) and the highest tertile of the 10-mile metric (OR 1.34; CI 1.04–1.72). Little indication of an association with SGA or term birthweight was found.

Analysis of cancer risk showed an increased incidence of urinary bladder cancer in females and males over time (Finkel et al. 2016). Moreover, thyroid cancer in females and males and leukemia increased over the examined time periods in all counties; however, this increase occurred regardless of HF activities. The incidence of childhood leukemia was reported to be increased by more than fourfold for the age group of 5–24 years in a rural region of Colorado (McKenzie et al. 2017). However, no increase in childhood leukemia was obtained for children up to 4 years. Additionally, non-Hodgkin lymphoma was not increased in children or adults.

In the 15 studies, mostly indirect measures of exposure were used, such as the number of gas wells or distance between places of residence and production sites. Exceptions were the occupational study of Esswein et al. (2014) and the public health study of Steinzor et al. (2013) which also reported data of airborne exposure, groundwater tests and inner exposure. These will be described in more detail below. Steinzor et al. (2013) performed a self-reporting health survey and environmental testing project between August 2011 and July 2012 that involved 108 individuals in 55 households in 14 counties across Pennsylvania. For 18 of the 20 symptoms (i.e., sinus problems, nasal irritation, increased fatigue, feeling weak and tired, joint pain, and shortness of breath), a higher percentage of those living within 1500 feet of a gas extraction and production facility experienced the symptom than of those living further away. Furthermore, a total of 34 air tests with a 24 h sampling time and nine water tests were conducted at 35 households. A total of 19 VOCs were detected in the ambient air sampled outside homes. The maximum concentration of ‘Total Hydrocarbons’ was 146 µg/m^3^; with benzene, toluene, ethylbenzene and *o*-xylene and the sum of *m*-/*p*-xylene reaching maxima of 1.5 µg/m^3^, 7.9 µg/m^3^, 1.5 µg/m^3^, 1.9 µg/m^3^ and 5.2 µg/m^3^, respectively. Among the halogenated hydrocarbons analyzed, methylene chloride ranked highest, with a maximum concentration of 32.62 µg/m^3^, followed by tetrachloroethylene and trichloroethylene, with maxima of 10.85 µg/m^3^ and 5.37 µg/m^3^, respectively. Maximum concentrations of the ketones 2-butanone and acetone were 2.9 and 19 µg/m^3^. Iron, manganese, arsenic, and lead were detected in the nine water well samples at levels that partly exceeded drinking water MCLs. The authors discussed associations between the chemicals measured in air and water and the health symptoms reported by residents predominantly in a plausible qualitative manner. Many of the chemicals quantitatively measured are known to be related to oil and gas operations and to the health symptoms. By contrast, the analyzed ambient air levels were, in part, below guidance values for acute and chronic exposure and due to the single 24 h sampling only reflects a snap-shot of the effects. The origin of the chemicals contributing to the overall impairment to air and water can also come from sources other than HF. Esswein et al. (2014) investigated the exposure of workers during flow-back operations in unconventional oil and gas extraction using real-time measurements to characterize air peak concentrations in various workplace areas, especially for VOCs and benzene. Urinary *S*-phenyl mercapturic acid (S-PMA) was used as a marker for benzene inner body burden. Airborne concentrations of hydrocarbons, including benzene, fluctuate greatly. Benzene was identified as the primary VOC exposure hazard for workers. Full-shift personal breathing zone benzene samples [time-weighted average (TWA)] from four different sites ranged from 0.007 to 0.59, 0.11 to 0.17, 0.02 to 0.50, and 0.004 to 0.02 ppm, respectively, and partly exceeded NIOSH’s OEL (occupational exposure limit) of 0.1 ppm benzene (Recommended Exposure Limit, REL-TWA). The arithmetic mean of urinary S-PMA from workers performing tank gauging was 6.5 µg/g creatinine (SD 5.5 μg/g creatinine). By contrast, the arithmetic mean of S-PMA in urine from workers not gauging tanks was 3.1 μg/g creatinine (SD 3.7 μg/g creatinine). Although sample numbers were limited and no correction for smoking was made, S-PMA in the urine of workers was moderately correlated with full-shift personal breathing zone benzene TWA concentrations (*r* = 0.56). While detectable concentrations of S-PMA were measurable in the urine of workers, none of the samples exceeded the ACGIH Biological Exposure Index (BEI) of S-PMA of 25 μg/g creatinine.

A systematic review of the existing epidemiologic literature on potential adverse health outcomes in populations living near oil and natural gas operations (ONGs) in the USA was performed by Bamber et al. (2019). The authors defined ONGs (or development) to include all upstream processes involved in the extraction of ONG resources using any combination of vertical drilling, directional/horizontal drilling, and hydraulic fracturing to access oil and natural gas from conventional and unconventional geologic formations. The evaluation of key studies to determine the level of certainty was based on 14 questions relating to population and sample, exposure, health outcomes, confounders, and reporting. Study findings were rated as having low, moderate, or high certainty that the estimated effect was close to that of the true effect. Among the 20 research articles, the level of certainty of four studies was rated as moderate and the level of all others as low. For each health outcome, weight-of-evidence levels were determined as substantial, moderate, limited, mixed, failing to show an association, or insufficient. The weight-of-evidence for studies on birth defects was assessed as insufficient, but as mixed for the birth outcomes decreased term birth weight or low birth weight, low APGAR score, preterm/premature birth, and small for gestational age; whereas, early infant mortality, fetal death, gestation period, and low infant health index were assessed as insufficient. The weight-of-evidence for the different cancer endpoints was overwhelmingly rated as insufficient (non-Hodgkin’s lymphoma (childhood), CNS tumors (child), urinary bladder, thyroid, and leukemia). The exception was leukemia (childhood non-specific and acute lymphocytic leukemia), for which the evidence was mixed. The weight-of-evidence was also assessed as mixed for the health outcomes: cardiovascular (hospitalizations), dermal (self-reported symptoms), psychological (self-reported symptoms), and respiratory (self-reported symptoms, hospitalizations). By contrast, self-reported cardiovascular symptoms, neurological (hospitalizations), psychological (diagnosed sleep disturbances), and “Others” (all hospitalizations) have been evaluated as insufficient. Self-reported gastrointestinal symptoms and self-reported neurological symptoms were classified as limited—failing to show an association. The authors concluded that the 20 studies with 32 different health outcomes of residents living near ONG operations analyzed in the review provide limited evidence (modest scientific findings that support the outcome, but with significant limitations) of harmful health effects, including asthma exacerbations and various self-reported symptoms. For all other health outcomes, conflicting evidence (mixed), insufficient evidence, or in some cases, a lack of evidence of the possibility for harmful health effects have been found. A summary of key messages of epidemiological research articles considered in the above-mentioned review articles is given in Table 13.

**Table 13:** Associations between health effects and HF exposure

Authors (year)	Study type	Population	Health outcome	Results
Hill (2013)*	Cross-sectional	Mothers living near a completed gas site versus a future gas site in Pennsylvania; *n* = 22,257 births from 2003 to 2010	Birth outcomes	Positive associations with lower birth weights, APGAR scores and small for gestational age
Fryzek et al. (2013)*^#^	Ecological	Children with cancer before and after oil/gas drilling in Pennsylvania from 1990 to 2009; Leukemia before/after drilling *n* = 457/*n* = 471 cases CNS tumors before/after drilling *n* = 327/*n* = 392 cases	Childhood cancer	No evidence of a relationship between increasing incidence of cancers and the number of wells or the type of wells; number of all cancers before/after drilling: SIR = 0.94; 95% CI 0.90–0.99/SIR = 1.02; 95% CI 0.98–1.07; childhood leukemia before/after drilling: SIR = 0.97; 95% CI 0.88–1.06/SIR = 1.01; 95% CI 0.92–1.11; CNS tumors after drilling were slightly elevated (OR 1.13, 95% CI 1.02–1.25)
Steinzor et al. (2013)*^#^	Cross-sectional	Survey of residents in Pennsylvania; from August 2011 and July 2012; *n* = 108	Self-reported symptoms of sinus/respiratory effects; skin, eye, nose, and throat irritation, neurological symptoms	Significant throat/nasal irritation, sinus problems, eye burning, headaches, skin rashes, loss of sense of smell, persistent cough, frequent nose bleeds, swollen painful joints; within a distance of 1.500 ft from nearest facility; participants (%) reported symptoms: sinus/respiratory (88), behavioral/mood/energy (74), neurological (80), muscles/joints (70), digestive/stomach (64), ear/nose/mouth (66), skin reactions (64), vision/eyes (63)
Esswein et al. (2014)	Cross-sectional	Workers in unconventional oil and gas extraction exposed during flow-back operations in spring and summer 2013 in Colorado and Wyoming	Burden of urinary s-phenyl mercapturic acid (s-PMA)	6.5 µg/g creatinine s-PMA (arithmetic mean) from workers performing tank gauging < 25 μg/g creatinine s-PMA (ACGIH BEI)
McKenzie et al. (2014)*^#^	Retrospective cohort	Mothers living within various densities of a well site between 1996 and 2009 in Colorado; *n* = 124,842 births	Birth outcomes	Prevalence of infant congenital heart defects increased with exposure tertile (OR 1.3 (highest tertile), 95% CI 1.2, 1.5), neural tube defects prevalence was associated with the highest tertile of exposure (OR 2.0; 95% CI 1.0, 3.9, based on 59 cases); exposure was negatively associated with preterm birth and slightly positively associated with fetal growth, no association was found between exposure and oral clefts
Saberi et al. (2014)	Cross-sectional	Pennsylvania Marcellus Shale region residents; one week during summer 2012, *n* = 72	By interview self-reported medical symptoms of sinus/respiratory effects; skin, eye, nose, and throat irritation; neurological effects	16 of 72 participants (22.2%) perceived “Natural gas activity” as a health concern and 9 participants (12.5%) attributed medical symptoms to HF exposures
Jemielita et al. (2015)*^#^	Ecological	Patients (inpatient hospitalization) in Pennsylvania from 2007 to 2011; *n* = 92,805	Hospitalization rates	Increases in cardiac and neurology admissions; cardiology inpatient prevalence rates were significantly associated with number of wells per zip code (*p* < 0.00096) and wells per km2 (*p* < 0.00096) while neurology inpatient prevalence rates were significantly associated with wells per km2 (*p* < 0.00096)
Rabinowitz et al. (2015)*^#^	Cross-sectional	Survey of residents in Pennsylvania during summer 2012; *n* = 492	Self-reported symptoms of respiratory effects and skin, eye, nose, and throat irritation; gastrointestinal, cardiovascular, and neurological symptoms	Positive associations with self-reported skin conditions and upper respiratory symptoms; no associations seen with lower respiratory, cardiac, gastrointestinal, or neurologic self- reported symptoms; the number of self-reported health symptoms per person was higher for residents living < 1 km (mean ± SD, 3.27 ± 3.72) compared with > 2 km from the nearest gas well (mean ± SD, 1.60 ± 2.14; *p* = 0.0002); upper respiratory symptoms were more frequently reported in persons living < 1 km from gas wells (39%) compared with households 1–2 km or > 2 km from the nearest well (31 and 18%, respectively) (*p* = 0.004); self-reported skin conditions were more common for < 1 km compared with > 2 km from the nearest gas well (odds ratio = 4.1; 95% CI 1.4, 12.3; *p* = 0.01).
Stacy et al. (2015)*^##^	Retrospective cohort	Mothers living within various densities of a well site in Pennsylvania from 2007 to 2010; *n* = 15,451 births	Birth outcomes	No evidence of premature births; comparison of the most to least exposed, revealed lower birth weight (3323 ± 558 vs 3344 ± 544 g) and a higher incidence of small for gestational age (6.5 vs 4.8%, respectively; odds ratio 1.34; 95% CI 1.10–1.63
Casey et al. (2016)**^##^	Retrospective cohort	Mothers living within various proximities of a gas development site in Pennsylvania from January 2009 to January 2013. *n* = 9384 mothers, who delivered 10,496 neonates	Birth outcomes	Increased premature births; association between HF activity and preterm birth that increased across quartiles, with a fourth quartile odds ratio of 1.4 (95% CI 1.0–1.9)
Finkel (2016)^#^	Ecological	Residents in Southwest Pennsylvania between 2000 and 2012, *n* = 1041,169 (as of 2010)	Cancer incidence (urinary bladder, thyroid and leukemia)	Number of urinary bladder cases was higher than expected in both sexes in counties with shale gas activity; SIR: 1.21, 95% CI 1.19–1.23 (male); SIR: 1.20, 95% CI 1.17–1.23 (female), both 2008–2012
Ma et al. (2016)^#^	Interrupted time series	2003 to 2012 births in Pennsylvania, *n* = 1401,813 births were included	Birth defects	birth defects prevalence rate was 6.3/1000 live births before HF and 5.0/1000 live births after HF (*p* < 0,01); the adjusted OR for annual post-HF birth defects trend was 1.00 (*p* = 0.29) and post-HF birth defects level decreased but not statistically significant (OR 0.97, *p* = 0.12); ORs for HF drilling well density per km2 on birth defects was 0.93 (*p* = 0.10)
Rasmussen et al. (2016)**^##^	Nested case–control	Asthma patients living within various metrics of oil and gas operation (pad preparation, drilling, stimulation, and production) in Pennsylvania from 2005 to 2012; *n* = 35,508	Respiratory effects	Positive associations among all levels of asthma exacerbation and all stages of HF; ORs (95% CI) ranged from 1.5 (1.2–1.7) for the association of the pad metric with severe exacerbations to 4.4 (3.8–5.2) for the association of the production metric with mild exacerbations
Werner et al. (2016)*	Ecological	Coal seam gas population, coal mining area population, and a rural/agricultural area population in Queensland from 1995 to 2011; *n* = 238,460 (CSG area)	Hospitalization rates	No significant increases in all-cause hospitalization rates over time for all- ages; the CSG area did not have significant increases in all-cause hospitalization rates over time for all-ages compared to the coal and rural study areas in adjusted models (RR: 1.02, 95% CI 1.00–1.04 compared to the coal mining area; RR: 1.01, 95% CI 0.99 –1.04 as compared to the rural area); CSG area showed increased hospitalization rates compared only to the rural area for neoplasms (RR: 1.09, 95% CI 1.02–1.16) and blood/immune diseases (RR: 1.14, 95% CI 1.02–1.27)
Busby and Mangano (2017)^#^	Ecological	Ten counties in Pennsylvania most affected by HF; *n* = 82,558 births	Early (0–28 days) infant mortality	Comparison of early infant mortality for 2007–2010 after HF development with a control period 2003–2006 showed in the 10 fracked counties a significant increase in mortality (238 vs 193; RR = 1.29; 95% CI 1.05, 1.55; *p* = 0.011).
Currie et al. (2017)^#^	Retrospective cohort	*n* ≈ 1.1 million births in Pennsylvania from 2004 to 2013	Birth outcomes	Greater incidence of low–birth weight babies, significant declines in average birth weight, and in an index of infant health for mothers living within 3 km from frac sites
McKenzie et al. (2017)*^#^	Case–control	Children living within various densities of oil and gas wells in Colorado; cancer diagnosis between 2001 and 2013; *n* = 137 cases	Childhood cancer (ALL, NHL)	Positive association with childhood acute lymphocytic leukemia, for ages 5–24, ALL cases were 4.3 times as likely to live in the highest tertile, compared to controls (OR 4.3, 95% CI 1.1–16), no association with non-Hodgkin’s lymphoma
Tustin et al. (2017)*^#^	Cross-sectional	Survey of patients/residents in Pennsylvania in early 2014; *n* = 7785	Upper respiratory and neurological	HF is associated with chronic rhinosinusitis, migraine headache, and fatigue symptoms in a general population; adjusted ORs for the highest quartile of HF activity compared with the lowest were 1.49 (95% CI 0.78–2.85) for CRS plus migraine, 1.88 (95% CI 1.08–3.25) for CRS plus fatigue, 1.95 (95% CI 1.18–3.21) for migraine plus fatigue, and 1.84 (95% CI 1.08, 3.14) for all three outcomes
Weinberger et al. (2017)	Cross-sectional	Adults who lived within 1 km of a well in Pennsylvania between; February 2012-October 2015; *n* = 51	By interview self-reported symptoms of sinus/respiratory effects; skin, eye, nose, and throat irritation; neurological effects	Reported symptoms (> 20% of sample) were sleep disturbance, headache, throat irritation, stress/anxiety, cough, shortness of breath, sinus, fatigue, wheezing, nausea; number of symptoms/participant ranged from 0 to 19 (mean 6.2; SD 5.1)
Casey et al. (2018)^#^	Case–control, cross-sectional	Participants in Pennsylvania between 2014 and 2015 and 2009–2015; *n* = 4762	Depression symptoms, disordered sleep	High HF (vs. very low) was associated with depression symptoms in an adjusted negative binomial model ( exponentiated coefficient = 1.18, 95% CI 1.04–1.34)
Elliott et al. (2018)^#^	Cross-sectional	Residents in Ohio, June–August 2016; *n* = 66	Self-reported health symptoms	General symptoms (stress, fatigue, muscle or joint pain, any other health symptoms); (adjusted OR 1.52, 95% CI 1.02, 2.26)
Hill (2018)^#^#	Retrospective cohort	Mothers in Pennsylvania from 2003 to 2010	Birth outcomes	Increased low birth weight and decreased term birth weight on average, small for gestational age and APGAR scores < 8 among mothers living within 2.5 km of a well
Peng et al. (2018)^#^	Ecological	Patients (inpatient hospital admissions) in Pennsylvania between 2001 and 2013	Acute myocardial infarction, chronic obstructive pulmonary disease, asthma, pneumonia, and upper respiratory infections	Significant association between HF and hospitalizations for pneumonia among the elderly
Whitworth et al. (2017)^#^	Retrospective cohort	158,894 women in North Texas with a birth or fetal death, November 2010–November 2012;	Preterm birth, small for gestational age, fetal death, birthweight	Increased adjusted odds of preterm birth in the highest tertiles of the 1/2- (OR 1.14; 95% CI 1.03–1.25), 2- (OR 1.14; 95% CI 1.07–1.22), and 10-mile (OR 1.15; 95% CI 1.08–1.22) metrics; increased adjusted odds of fetal death in the second tertile of the 2-mile metric (OR 1.56; 1.16–2.11) and the highest tertile of the 10-mile metric (OR 1.34; 95% CI 1.04–1.72)
Whitworth et al. (2018)^#^	Case–control	Women with singleton births in Texas; *n* = 13,328	Preterm birth	Increased ORs of preterm birth in the third tertile of the HF drilling (OR 1.20, 95% CI 1.06–1.37) and HF-production (OR 1.15, 95% CI 1.05–1.26) metrics
Casey et al. (2019)	Retrospective cohort	Births to 7715 mothers in Pennsylvania between 01/2009 and 01/2013 *n* = 8371	Birth outcomes	4.3 additional cases of antenatal anxiety or depression per 100 women (95% CI 1.5, 7.0) for mothers lived in the highest quartile of HF activity vs. quartiles 1–3
Denham et al. (2019)	Ecological	Hospitalizations in 67 Pennsylvania counties and 54 counties that are not large metropolitan between 2003 and 2014	Primary diagnosis according to 16 ICD-9-CM major categories	Positive associations between genitourinary hospitalizations and cumulative well density; positive associations between skin-related hospitalizations and cumulative HF measures
McKenzie et al. (2019)	Case–control	Infants born in Colorado between 2005 and 2011; *n* = 3324	Birth outcomes	Congenital heart defects were 1.4 (1.0–2.0) (OR, 95% CI) and 1.7 (1.1–2.6) times more likely than controls in the medium and high intensity exposure groups, compared to the low intensity group; pulmonary artery and valve defects were 1.7 (0.93–3.0) and 2.5 (1.1, 5.3) times more likely in the

Quality rating * (low) and ** (medium) corresponds to the detailed evaluation of the study quality according to CDEHP (2017), Appendix 2C (individual study evaluations), and Appendix 2A (systematic review methodology)

Level of certainty # (low) and ## (moderate) in accordance with Bamber et al. (2019)

In principle, it could be expected that adverse health effects due to HF are more severe compared to conventional gas and oil production since HF can cause exposure to a higher number of toxic compounds, including those exhibiting endocrine activity (Balise et al. 2016). Therefore, the quality of exposure assessment is crucial. Most exposure estimates in epidemiological studies on HF are based on sophisticated but indirect distance measurements or HF activity metrics and not on measured contaminant concentrations in ambient/indoor air, soil, groundwater, and drinking water. A recent study compared exposure categories based on ambient air measurements between 2011 and 2015 and estimates of distance-based well activity metrics for each phase of well development (pad preparation, drilling, fracturing, and production) (Wendt Hess et al. 2019). Daily mean air monitoring data for benzene, carbon monoxide, nitrogen dioxide, ozone, fine particulates and sulfur dioxide were combined with data on 8885 wells in Pennsylvania. Ambient air samples of the six pollutants were collected at 76 monitoring sites. The results suggest that the well activity metrics do not adequately distinguish categories of air pollutant exposure and calculated exposure estimates did not agree with those from air sampling data. Using distance-based well activity metrics as surrogate for ambient air exposure can result in misclassification.

Apart from general limitations in terms of bias (including confounding), use of human studies is complicated due to the occurrence of other co-exposures and the fact that unexposed individuals usually do not exist. The variability in terms of susceptibility to chemical exposures and interaction with other lifestyle factors means that results from different epidemiological studies can be conflicting (Lanzoni et al. 2019). However, epidemiological studies of sufficient size and quality that include precise exposure monitoring are not yet available. It has been concluded that there is an urgent need for high-quality epidemiological studies to assess possible adverse health effects of HF (Rabinowitz et al. 2015; Saunders et al. 2018; SCHEER 2018; Wright and Muma 2018; Bamber et al. 2019).

## Epidemiological studies in regions with unconventional oil and gas production in Germany

Suspected local cancer clusters in the neighborhood of natural gas or oil production facilities with former frac operations (LBEG 2019b) prompted advanced cancer cluster investigations in several regions of Lower Saxony. The focus lay on hematological malignancies according to ICD-10 (C81–C96) (EKN 2014, 2015, 2016; LK Rotenburg 2017). A statistically significant increase in the incidence of leukemia and lymphoma (ICD-10 C81–C96) was observed in men of the joint community of Bothel in the district of Rotenburg; 41 cases were observed and 21.3 expected (SIR: 1.93; 95% CI 1.38–2.61) (EKN 2014). An analysis of sub-groups of leukemia and lymphoma (C81–C96) showed the strongest increase for multiple myelomas (C90), non-Hodgkin lymphomas (C82–C85), followed by leukemias (C91–C95) and Hodgkin lymphomas (C81). In contrast to men, only 15 leukemia and lymphoma cases were observed for women. With 16.8 expected cases, this did not represent a statistically significant result (SIR: 0.89; 95% CI 0.50–1.47).

In a follow-up study, 6978 inhabitants of the joint community Bothel were invited to be interviewed concerning their own hematological diseases and hematological diseases of relatives based on an environmental–medical questionnaire. The response rates ranged between 69.3 and 61.2% in the individual communes. In total, 37 validated incident cases in men occurred between 1997 and 2015. During statistical evaluation, an indication was obtained for an increased cancer incidence for individuals employed in the wood-processing industry. Case–control analyses were performed for (a) all 37 hematological cancer cases (between 1997 and 2015), (b) the 26 cases with non-Hodgkin lymphoma (1995–2015) and (c) only the 19 cases of non-Hodgkin lymphoma and multiple myeloma (MM) diagnosed between 2007 and 2015. Using these comparisons, a possible association was studied between hematological cancer cases in men and the distance of the place of residence to the following potential sources of emission: (a) gas production site, (b) sludge pits, (c) wood-processing companies, (d) metal-processing companies, (e) petrol stations, (f) agricultural trading stations, (g) garden centers, (h) former railway lines due to the use of pesticides. These analyses resulted only in an indication of a possible association between the distance of residence to drilling fluid pits and hematological cancer cases in men.

The observation of increased hematological cancer cases in the first study of the joint community Bothel (EKN 2014) triggered an extended study including seven communities east and west of the joint community Bothel and the city of Rotenburg located northwest of Bothel (EKN 2015). The communities were grouped into three regions, A–C, and the risk of hematological cancer incidence between 2003 and 2012 was analyzed (EKN 2015). A statistically significantly increased cancer incidence for leukemia and MM was obtained for the city of Rotenburg (region B) with 72 observed, compared to 54.8 expected cancer cases (SIR: 1.31; 95% CI 1.03–1.66) only for men. By contrast, no statistically significant difference was obtained for women, with 53 observed and 48.3 expected cancer cases (SIR: 1.10; 95% CI 0.82–1.43). The strongest increase for men was obtained for MM. No significant increase in cancer incidence was observed for regions A and C (EKN 2015). It is striking that a similar pattern of increased cancer incidence was observed for the city of Rotenburg and the joint community of Bothel, but not for the other regions.

A further study was performed in the joint community Steimbke in Lower Saxony (EKN 2016), triggered by evidence of a possibly increased incidence of childhood leukemia in one of the local communities (Rodewald). Of the 46 hematological cancer cases registered between 2005 and 2013 in the joint community Steimbke, 19 occurred directly in the community Rodewald and further 24 cases in other communities of this region. However, for three of the 46 cases, no information was available on the exact place of residence. Summarizing all cases of males and females, 46 hematological cancer cases were observed, compared to 36.2 expected (SIR: 1.27; 95% CI 0.93–1.69). Considering the community Rodewald alone, 20 incident cases were observed, compared to 12.7 expected (SIR: 1.27; 95% CI 0.93–1.69). Both SIR were statistically not significant; however, statistical significance would have been reached if two of the three cases for whom no information on the place of residence was available, would have been assigned to the community of Rodewald.

In parallel, the German Childhood Cancer Registry performed an evaluation of incidental hematological cancer cases in children aged < 15 years in the region’s joint community of Steimbke and the community Rodewald (EKN 2016). In the observation period between 1987 and 2014, six cases of childhood leukemia were observed, compared to 1.7 expected (SIR: 3.6; 95% CI 1.3–7.8). The place of residence of four cases was Rodewald, where only 0.6 cases were expected (SIR: 6.8; 95% CI 1.9–17.4). Therefore, a statistically significantly increased incidence of childhood leukemia in the joint community Steimbke and the community Rodewald was confirmed. Moreover, the temporal clustering was evident because, between 2004 and 2007, three incident cases were already observed in Rodewald and two further in Steimbke. A structured interview by means of a medical questionnaire of the six cancer patients revealed no evidence of relevant commonalities (Nienburg 2017). The toxicological analysis of environmental pollution of the former production site, the extent of expected additional and total emissions, and the increased cases of childhood leukemia lead to a plausible scenario of exposure and adverse health effects. It should be considered that regional clusters of childhood leukemia and lymphoma represent a global, not yet fully understood, phenomenon (Grosche et al. 2017).

The above-described statistically significant associations led to the question of whether proximity of the place of residence to oil or gas production sites or to sludge pits is generally associated with an increased incidence of hematological cancer (ICD 10, C81–C96) in Lower Saxony. To answer this question, a comprehensive case–control study was performed (Forster et al. 2018). For this purpose, 3978 hematological cancer cases registered in the Epidemiological Cancer Register of Lower Saxony were compared to 15,912 controls (no cancer) that were randomly chosen from the register of residents. As a measure of exposure, the 1 km radius of the place of residence of cases and controls to (a) oil or gas production sites (*n* = 637) and (b) sludge pitches (*n* = 493) were chosen. To analyze a possible association between exposure (1 km radius) and cancer cases, logistic regression models were used which were adjusted for the confounders ‘proximity of residence to main roads’ and ‘proximity of residence to agricultural areas’. Further confounders were not considered. No significant association was obtained for the two main hypotheses: proximity to production sites and incidence of hematological cancer (OR 0.98; 95% CI 0.85–1.13), or proximity to sludge pits and incidence of hematological cancer (OR 0.97; 95% CI 0.81–1.17). There were no differences observed between males and females. Moreover, both exposure measures were also not associated with further cancer entities, such as non-Hodgkin lymphoma, MM or acute myeloid lymphoma. The above-described significant association of an increased incidence of hematological cancer for Rotenburg identified in 2014 was also observed in the comprehensive case–control study; however, no significant association was obtained when the entire region was considered. In conclusion, comprehensive epidemiological studies in Lower Saxony showed several local associations with an increased cancer risk; however, this was not confirmed when all regions with proximity to production sites in Lower Saxony were considered. Therefore, the comprehensive studies did not lead to the establishment of a causal relationship of oil and gas production in Lower Saxony and increased cancer risk.

### Human biomonitoring studies (HBM)

Only a few studies analyzed internal exposure to HF-related chemicals. Caron-Beaudoin et al. (2018) evaluated gestational exposure to benzene and toluene as known developmental toxicants in the Peace River Valley, Northeastern British Columbia (Canada). Metabolites of benzene [*S*-phenylmercapturic acid (S-PMA) and trans,trans-muconic acid (t,t-MA)], and toluene [*S*-benzylmercapturic acid (S-BMA)] were measured in pooled urine samples from 29 pregnant women who collected 12 mL urine samples over five consecutive days. The median sampling time was 9:00 PM, and ranged from 2:00 PM (10th percentile) to 11:00 PM (95th percentile). The median S-PMA level (0.18 μg/g creatinine) in this study was similar to that in the general Canadian population. However, the median t,t-MA level (180 μg/g creatinine) was approximately 3.5 times higher. Participants that reported exposure to cigarette smoke had median urinary S-PMA, t,t-MA and S-BMA levels of 0.21, 202 and 6.88 μg/g creatinine, respectively. Participants with exposure from the workplace (*n* = 6) such as mining industry, natural gas, construction, forestry, pipeline maintenance or at hydroelectric dams had median urinary S-PMA, t,t-MA and S-BMA levels of 0.23, 347 and 4.31 μg/g creatinine, respectively. The median urinary level of S-BMA in the participants correlated well with the median of 7.2 μg/g creatinine from female Americans that participated in the National Health and Nutrition Examination Survey (NHANES). The observed t,t-MA levels may be due to sorbic acid, a food preservative partially metabolized into t,t-MA. Further limitations of the study are the small numbers of participants and not using 24-h urine samples.

The confirmed cluster of hematological malignancies in a residential population adjacent to natural gas fields (NGFs) in Lower Saxony (Germany) triggered the conduct an HBM study to determine the current internal and external exposure of residents near NGFs to benzene (Göen et al. 2019, 2020). In total, 110 residents (73 non-smokers and 37 smokers) were recruited from the joint community, with NGFs (study group) and 84 residents (non-smokers only) of the same county without NGFs (controls, COs). Probands collected 24-h urine samples over two consecutive workdays and two non-workdays within 14 days from 12/07/18 to 30/07/18 (SG), and from 29/10/18 to 16/11/18 (study group and controls). S-PMA excretion in the study group was higher for smokers (median 2.33 μg/g creatinine, range 0.10–25.2 μg/g creatinine, *n* = 37) than for non-smokers (median 0.11 μg/g creatinine, range 0.05–1.17 μg/g creatinine, *n* = 65) during sampling in the fall. However, there was no difference between non-smokers of the potentially exposed study group and controls (median 0.12 μg/g creatinine, range 0.03–0.72/g creatinine, *n* = 78). S-PMA levels in the study group were higher in fall when compared to sampling in summer [non-smokers: median 0.11 μg/g creatinine (*n* = 65) vs. 0.05 μg/g creatinine (*n* = 66); smokers: median 2.33 μg/g creatinine vs. 1.51 μg/g creatinine, both *n* = 37]. Median benzene air levels were 1.03, 1.09, and 0.50 μg/m^3^ for personalized, indoor air, and ambient air samples for non-smokers in fall.

In response to citizens’ concerns living in and near the town of DISH (Barnett Shale), the Texas Department of State Health Services (TxDSHS) conducted an HBM study collecting blood and urine samples (first morning void) from 28 people. Blood samples were analyzed for a wide range of VOCs. Although several VOCs were detected in some of the blood samples (1,2-dichloroethane; tetrachloroethene; bromoform; benzene; chloroform; dibromochloromethane; 1,4-dichlorobenzene; ethylbenzene; *o*-xylene; styrene; trichloroethene; 1,1,1-trichloroethane; toluene, and *m*-/*p*-xylene), the pattern of VOC values was not consistent with a community-wide exposure to airborne contaminants, such as those that might be associated with natural gas drilling operations. Some individuals showed higher blood levels of bromoform (*n* = 3), chloroform (*n* = 10), and dibromochloromethane (*n* = 4) than 95% of the U.S. population. Other compounds that were found in a few individuals at levels higher than 95% of the general U.S. population included 1,2-dichloroethane, tetrachloroethene, 1,4-dichlorobenzene, trichloroethene, and 1,1,1-trichloroethane. Benzene levels in blood ranged from < LOD-0.027 (non-smokers) and 0.045–1.45 (smokers) µg/L and were detected in six individuals. Toluene, *m*-/*p*-xylene, and ethylbenzene were measured ranging from < LOD-3.25 µg/L (*n* = 18), < LOD-1.32 (*n* = 15), and < LOD-0.437 µg/L (*n* = 8), respectively. S-PMA ranged from < LOD-0.40 μg/g creatinine (non-smokers) and < LOD-2.79 μg/g creatinine (smokers). The DISH median values of benzene, toluene, and *m*-/*p*-xylene were not significantly different than the reference median from NHANES. Considering the short half-life of excretion of the investigated VOCs, the utilization of first morning void samples is critical.

## Summary

So far, the scientific investigation of possible health risks mediated by hydraulic fracturing operations has led to inconsistent results. The most critical part of risk assessment in this context is the exposure assessment which is hampered by the unavailability of data from qualified baseline monitoring before the start of frac operations. Hence, when assessing the HF impact on the environment and human health it is often difficult or practically impossible to estimate the proportion of HF which is contributing to the existing exposure. With regard to the origin of the emissions to be considered from HF and non-HF processes, differentiation must be made using suitable and specific target substances. They require adapted monitoring strategies and procedures for ground and surface waters as well as ambient air. Of all the sub-processes of HF operations, the fate and behavior of the production water and flow-back in the environment currently seems to be the greatest challenge. The complex pollutant inventories in the case of flow-back or production water are only approximately known. They are of particular importance if improper disposal of the production water can directly contaminate drinking water resources. The sustainable disposal of production water and flow-back or their reuse remains a challenge.

The use of realistic exposure scenarios, which are based on a strictly usage-based view of the subject of protection, is fundamental to the assessment of health risks from HF operations. Looking only/exclusively at the intrinsic toxicity of the frac chemicals cannot be conducive. Available epidemiological studies have shown significant associations between the emissions from HF processes and the observed health effects, but even large studies have not been able to prove a clear causality. Since in epidemiological studies human exposure is largely described using various distance measures as a surrogate, the challenge for future studies will be to use measured concentrations of pollutants. The fact that the exact composition of frac fluids and production water is partly not known (the recipes have only recently been published in relevant registers) can now be countered by analyzing the pollution in the subject of protection with an advanced range of chemical analytical methods. The use of frac chemicals with CMR properties remains problematic if it is still approved by governmental regulations. That in the past the toxicological database of used frac chemicals has been partially incomplete is a critical point in human risk assessment. In this case, for a sound characterization of the hazard of the relevant frac chemicals and human health risks, toxicological alternatives such as the precautionary-oriented TTC or HRIV approach should be used. The continuing utilization of the hydraulic fracturing technology requires compulsorily a well-founded contribution from toxicology with regard to the identification of possible hazard potentials of the relevant chemicals, the exposure characterization based on measured substance’s concentrations, and to the evaluation of health risks in relation to the general population.
